# Plasmid genomic epidemiology of *bla*_KPC_ carbapenemase-producing *Enterobacterales* in Canada, 2010–2021

**DOI:** 10.1128/aac.00860-23

**Published:** 2023-11-16

**Authors:** Nicole Lerminiaux, Robyn Mitchell, Jessica Bartoszko, Ian Davis, Chelsey Ellis, Ken Fakharuddin, Susy S. Hota, Kevin Katz, Pamela Kibsey, Jerome A. Leis, Yves Longtin, Allison McGeer, Jessica Minion, Michael Mulvey, Sonja Musto, Ewa Rajda, Stephanie W. Smith, Jocelyn A. Srigley, Kathryn N. Suh, Nisha Thampi, Jen Tomlinson, Titus Wong, Laura Mataseje, on behalf of the Canadian Nosocomial Infection Surveillance Program

**Affiliations:** 1 National Microbiology Laboratory, Winnipeg, Manitoba, Canada; 2 Public Health Agency of Canada, Ottawa, Ontario, Canada; 3 QEII Health Sciences Centre, Halifax, Nova Scotia, Canada; 4 The Moncton Hospital, Moncton, New Brunswick, Canada; 5 University Health Network, Toronto, Ontario, Canada; 6 North York General Hospital, Toronto, Ontario, Canada; 7 Royal Jubilee Hospital, Victoria, British Columbia, Canada; 8 Sunnybrook Health Sciences Centre, Toronto, Ontario, Canada; 9 Jewish General Hospital, Montréal, Québec, Canada; 10 Sinai Health, Toronto, Ontario, Canada; 11 Saskatchewan Health Authority, Regina, Saskatchewan, Canada; 12 Health Sciences Centre, Winnipeg, Manitoba, Canada; 13 McGill University Health Centre, Montréal, Québec, Canada; 14 University of Alberta Hospital, Edmonton, Alberta, Canada; 15 BC Women’s and BC Children’s Hospital, Vancouver, British Columbia, Canada; 16 The Ottawa Hospital, Ottawa, Ontario, Canada; 17 Children’s Hospital of Eastern Ontario, Ottawa, Ontario, Canada; 18 Vancouver Coastal Health Research Institute, Vancouver, British Columbia, Canada; Columbia University Irving Medical Center, New York, USA

**Keywords:** plasmid, carbapenemase, antimicrobial resistance, surveillance studies

## Abstract

Carbapenems are considered last-resort antibiotics for the treatment of infections caused by multidrug-resistant *Enterobacterales*, but carbapenem resistance due to acquisition of carbapenemase genes is a growing threat that has been reported worldwide. *Klebsiella pneumoniae* carbapenemase (*bla*_KPC_) is the most common type of carbapenemase in Canada and elsewhere; it can hydrolyze penicillins, cephalosporins, aztreonam, and carbapenems and is frequently found on mobile plasmids in the Tn*4401* transposon. This means that alongside clonal expansion, *bla*_KPC_ can disseminate through plasmid- and transposon-mediated horizontal gene transfer. We applied whole genome sequencing to characterize the molecular epidemiology of 829 *bla*_KPC_ carbapenemase-producing isolates collected by the Canadian Nosocomial Infection Surveillance Program from 2010 to 2021. Using a combination of short-read and long-read sequencing, we obtained 202 complete and circular *bla*_KPC_-encoding plasmids. Using MOB-suite, 10 major plasmid clusters were identified from this data set which represented 87% (175/202) of the Canadian *bla*_KPC_-encoding plasmids. We further estimated the genomic location of incomplete *bla*_KPC_-encoding contigs and predicted a plasmid cluster for 95% (603/635) of these. We identified different patterns of carbapenemase mobilization across Canada related to different plasmid clusters, including clonal transmission of IncF-type plasmids (108/829, 13%) in *K. pneumoniae* clonal complex 258 and novel repE(pEh60-7) plasmids (44/829, 5%) in *Enterobacter hormaechei* ST316, and horizontal transmission of IncL/M (142/829, 17%) and IncN-type plasmids (149/829, 18%) across multiple genera. Our findings highlight the diversity of *bla*_KPC_ genomic loci and indicate that multiple, distinct plasmid clusters have contributed to *bla*_KPC_ spread and persistence in Canada.

## INTRODUCTION

Carbapenems are considered last-resort antibiotics for the treatment of infections caused by multidrug-resistant Gram-negative bacteria. Following the use of carbapenems in clinical practice, the emergence of carbapenem-resistant pathogens poses a great threat to human health (1). Carbapenem-resistant *Enterobacterales* have been reported worldwide as a consequence largely of the acquisition of carbapenemase genes (2).

Of the different classes of carbapenemases, *Klebsiella pneumoniae* carbapenemase (*bla*_KPC_) is the most commonly identified in many countries including the United States and Canada [reviewed in references (3–6)]. *bla*_KPC_ was the dominant type of carbapenemase isolated from 2010 to 2014 by the Canadian Nosocomial Infection Surveillance Program (CNISP), where they comprised 50%–83% of all carbapenemases analyzed per year (6). From 2017 to 2021, the rates of carbapenemase-producing *Enterobacterales* infections in Canada increased by 166% (from *n* = 20 in 2017 to *n* = 55 in 2021), and *bla*_KPC_ remains the most common carbapenemase type detected in Canada (7).

Since the first identification of *bla*_KPC_ almost 30 years ago (8), *bla*_KPC_ has been found in over 100 different *K. pneumoniae* sequence types (STs) (9), but the original dissemination was driven primarily by the spread of *bla*_KPC_-producing *K. pneumoniae* isolates that are members of clonal complex 258 (9–12). Along with clonal expansion, *bla*_KPC_ can disseminate through plasmid- and transposon-mediated horizontal gene transfer and has been detected in at least 11 other genera (3, 10, 13–16).

Advances in genome sequencing have enabled detailed characterization of complete *bla*_KPC_-encoding plasmids in recent years (13, 15). *bla*_KPC_ is associated with a variety of plasmid types, including the narrow-host-range IncF-type plasmids which were crucial for the success of *K. pneumoniae* clonal complex 258 (3, 12, 15). *bla*_KPC_ has also been found on broad-host-range plasmids with IncN, IncR, IncX, ColRNA, IncA/C, or IncP replicons around the world (3, 13, 15, 17–20). Many of these plasmids can have multiple replicons, undergo large rearrangements, and encode other genetic features that ensure their persistence (20).

Here, we applied whole genome sequencing to characterize the molecular epidemiology of *bla*_KPC_ carbapenemase-producing isolates collected by the Canadian Nosocomial Infection Surveillance Program from 2010 to 2021. Using combined short-read and long-read sequencing of selected representatives to generate complete *bla*_KPC_-encoding plasmids, we investigated the diversity of carbapenemase-encoding plasmids among these isolates across Canada and compared them to the global context of *bla*_KPC_.

## RESULTS

### Characteristics of *bla*_KPC_ carbapenemase-producing isolates

**Fig 1:**
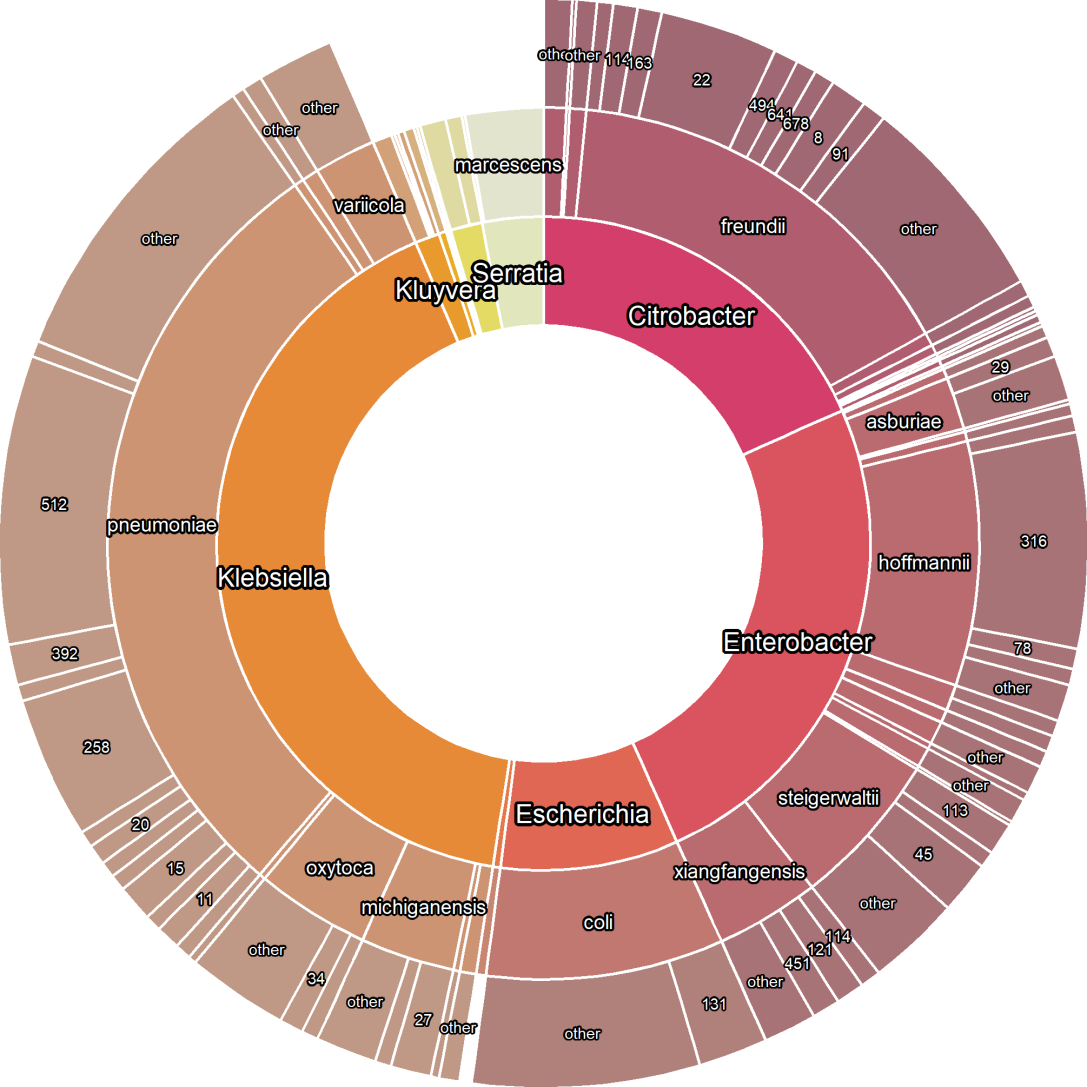
Summary of genera (inner ring), species (middle ring), and multi-locus sequence types (MLST; outer ring) of *bla*_KPC_-encoding isolates included in the study (829 total isolates). MLST profiles found in three or fewer isolates were grouped into “other”. Not all labels are displayed.

A total of 829 *bla*_KPC_-producing isolates were submitted by 34 hospital sites across Canada from 2010 to 2021. We performed short-read and long-read sequencing on a selection of 155 isolates. The *bla*_KPC_-producing isolates belonged to 10 genera and 26 different species, with the most common genera being the *Klebsiella pneumoniae* species complex (268/829, 32.3%), *Enterobacter cloacae* complex (203/829, 24.4%), *Citrobacter freundii* complex (132/829, 15.9%), and *Escherichia coli* (73/829, 8.8%) (Fig. 1). The most common sequence types were *K. pneumoniae* ST512 (71/268, 26.4%) and ST258 (35/268, 13.1%) (note that ST512 is a single locus variant of ST258), *E. cloacae* ST316 (53/203, 26.1%), *C. freundii* ST22 (29/132, 32.0%), and *E. coli* ST131 (17/73, 23.2%) (Fig. 1).

**Fig 2:**
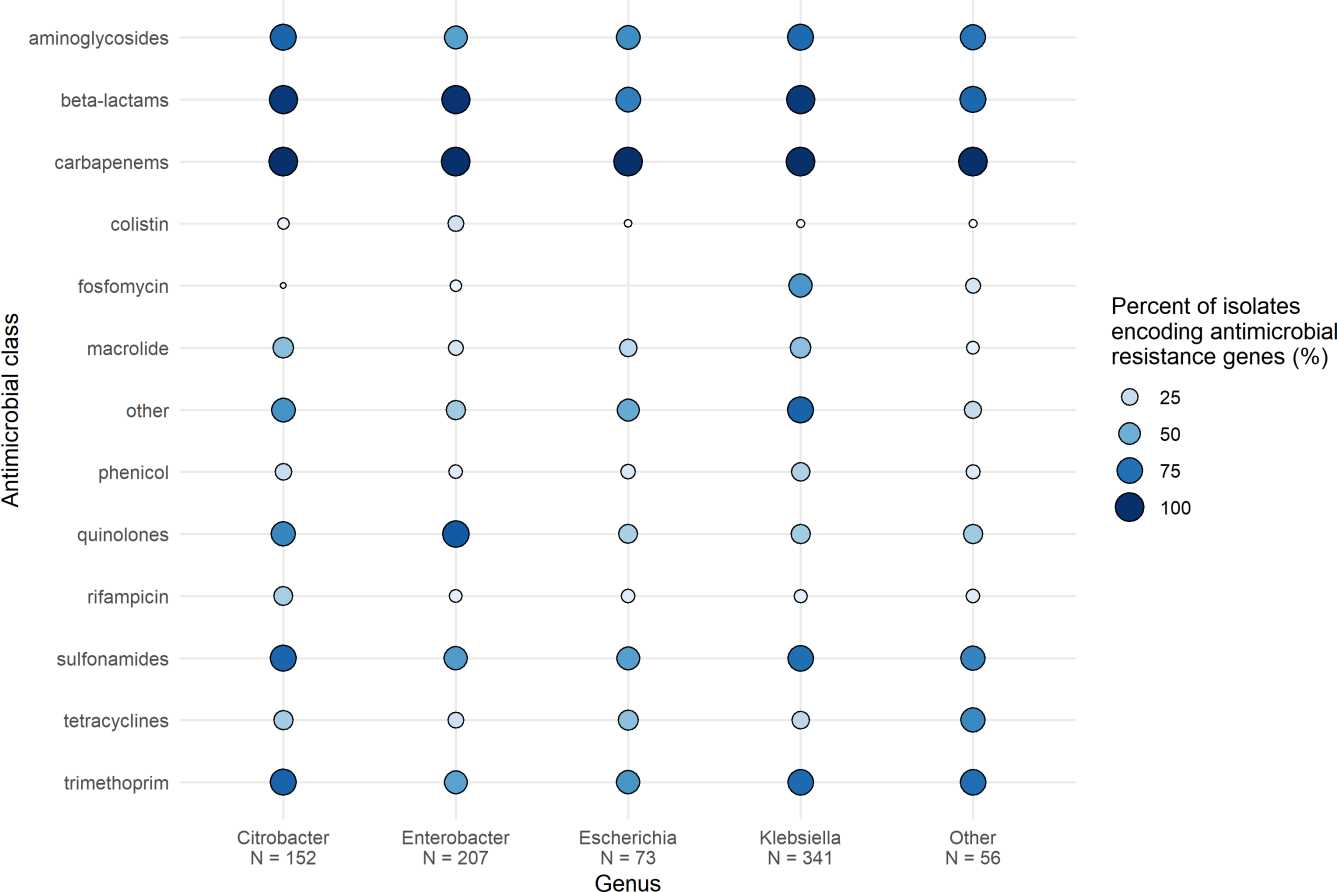
Proportion of isolates encoding antimicrobial resistance genes identified by StarAMR, then categorized by drug class is presented across the top four genera. Values represent proportion of isolates encoding genes belonging to a certain antimicrobial class. “*N*” indicates the number of isolates in that genus. The “Other” genera include *Serratia* spp., *Raoultella* spp., *Pseudescherichia* spp., *Pantoea* spp., *Morganella* spp., *Kluyvera* spp., and *Hafnia* spp.

Using the StarAMR tool for the detection of resistance genes in the whole genome sequencing data, we observed 93% (773/829) of *bla*_KPC_-producing isolates harbored additional β-lactamase genes alongside the *bla*_KPC_ carbapenemase (Fig. 2). Of the 773 isolates harboring additional β-lactamases, *bla*_TEM-1B_ (187/773, 24.2%), *bla*_OXA-9_ (146/773, 18.9%), *bla*_TEM-1A_ (125/773, 16.2%), *bla*_SHV-182_ (91/773, 11.8%), and *bla*_OXA-1_ (82/773, 10.6%) were the most common types. Aminoglycoside, sulfonamide, and trimethoprim resistance genes were commonly observed among multiple genera (Fig. 2); the most common genes included *sul1* (482/829, 58.1%), *qacE* (424/829, 51.1%), *aac*(6′)*-*Ib-cr (357/829, 43.1%), *sul2* (227/829, 27.4%), *aadA2* (225/829, 27.1%), *mphA* (224/829, 27.0%), and OqxA/B (224/829, 27.0%). β-Lactamases were significantly more likely to be found in *Enterobacter* spp., *Citrobacter* spp., or *Klebsiella* spp. than *E. coli* (*P* < 0.001 for all comparisons). Colistin and quinolone resistance genes were significantly more likely to be found in *Enterobacter* spp. than any other genera (*P* < 0.001 for all comparisons). Aminoglycoside, macrolide, sulfonamide, and trimethoprim resistance genes were significantly more likely to be found in *Klebsiella* spp. or *Citrobacter* spp. compared to *Enterobacter* spp. or *E. coli* (*P* < 0.035 for all comparisons). Rifampin resistance genes were significantly more likely to be found in *Citrobacter* spp. than any other genera (*P* < 0.001 for all comparisons).

A total of 844 *bla*_KPC_ genes were detected among the 829 isolates, indicating an occurrence of 1.7% of isolates harboring >1 copy of *bla*_KPC_. The *bla*_KPC-3_ variant was the most common (678/844, 80.3%) followed by *bla*_KPC-2_ (162/844, 19.2%). Two isolates had *bla*_KPC-4_, one isolate had *bla*_KPC-9_, and one isolate had *bla*_KPC-18_. In addition to *bla*_KPC_, several genomes carried additional carbapenemase genes (7/829, 0.8%; 2 *bla*_NDM-1_, 2 *bla*_NDM-5_, 1 *bla*_OXA-232_, and 2 *bla*_VIM-1_).

### Genomic context of *bla*_KPC_ genes

The *bla*_KPC_ genes are often located on the Tn*4401* transposon which facilitates their mobility to other DNA elements and strains (21, 22). In this data set, *bla*_KPC_ was frequently found within the previously described Tn*4401*b isoform (643/829, 77.6%), followed by the Tn*4401*a isoform (132/829, 15.9%), Tn*4401*d (5/829, 0.6%), and Tn*4401*e (1/829, 0.01%). The remainder of *bla*_KPC_ (38/829, 4.6%) were found on a combination of truncated Tn*4401*, a unique isoform of Tn*4401* or complete absence of Tn*4401*.

**Fig 3:**
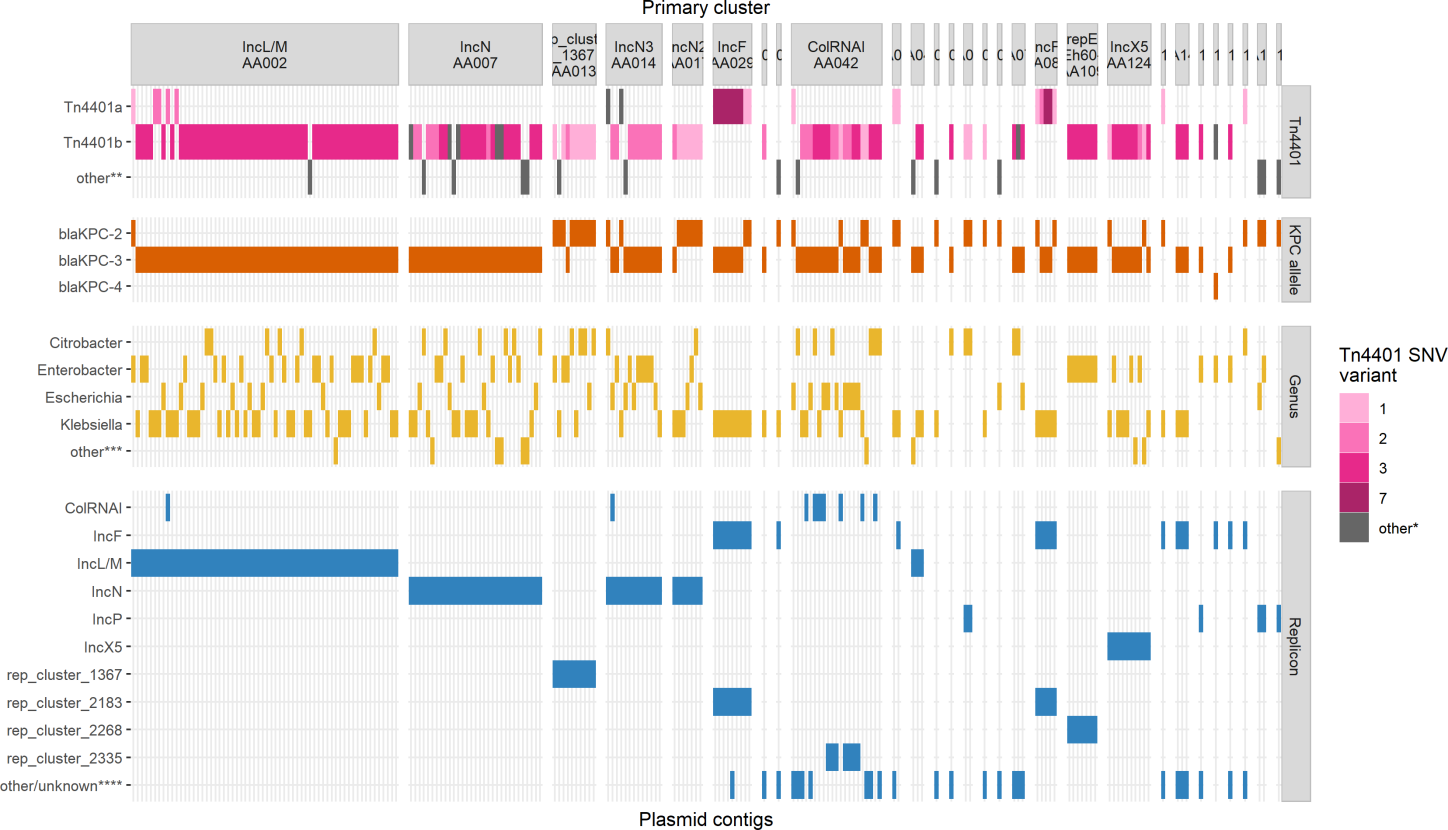
Characteristics of 202 *bla*_KPC_-encoding plasmids sequenced in this study. Groups on the *x*-axis correspond to primary cluster IDs generated among the plasmid database PLSDB and Canadian *bla*_KPC_-encoding plasmids. “Other*” indicates SNVs found in fewer than three plasmids or Tn*4401* absence. “Other**” indicates either truncated, alternative isoforms or absence of Tn*4401*. “Other***” indicates *Hafnia*, *Kluyvera*, *Morganella*, *Raoultella*, or *Serratia* genera. “Other/unknown****” indicates replicons (*n* = 26) found in fewer than five plasmids or replicons that could not be typed.

There were 25 different Tn*4401* isoforms in this data set. The majority of the single nucleotide variants (SNVs) within Tn*4401* observed here differed from those included in TETyper (23), so we generated a custom numbering scheme described in Table S2. The most common SNV profiles were 3 (439/829, 53.0%; A7029G|C8015T), 1 (128/829, 15.4%; none), 2 (115/829, 13.9%; C8015T, differentiates *bla*_KPC-2_ and *bla*_KPC-3_), 7 (56/829, 6.8%; C8015T|G9882T), 8 (18/829, 2.2%; A7029G|C7672T|C8015T), and 4 (10/829, 1.2%; A7029G|C8015T|T9317C) (Fig. 3).

### Complete and circular *bla*_KPC_ plasmids in a global context

Unsurprisingly, we obtained only 59 closed circular plasmids from Illumina-only assemblies. Using a hybrid approach on a subset of 155 isolates, we obtained an additional 143 complete circular plasmids. Complete *bla*_KPC_-encoding plasmids (*n* = 202) ranged from 7.6 kb to 374.7 kb in size and were distributed among the top species as described above (Fig. 3). Plasmid incompatibility groups included IncL/M (65/202, 32.2%), IncN types (51/202, 25.2%), IncF types (39/202, 19.3%), IncX5 (10/202, 5.0%), and ColRNAI types (21/202, 10.3%), among others. Plasmids were predicted to be conjugative (148/202, 73.2%), mobilizable (36/202, 17.8%), or non-mobilizable (18/202, 8.9%) based on the presence of self-encoded *oriT* sequences, relaxases, and mating pair formation (MPF) proteins detected by MOB-suite (24).

To investigate where these plasmids fit within a global data set, we clustered our 202 complete plasmids alongside 34,513 plasmids present in PLSDB (25) using the MOB-cluster tool from MOB-suite (24, 26). Altogether, the plasmids clustered into 10,996 distinct primary clusters, and our *bla*_KPC_ plasmids grouped into a subset of 28 primary clusters which all contained representative plasmids from PLSDB.

**Fig 4:**
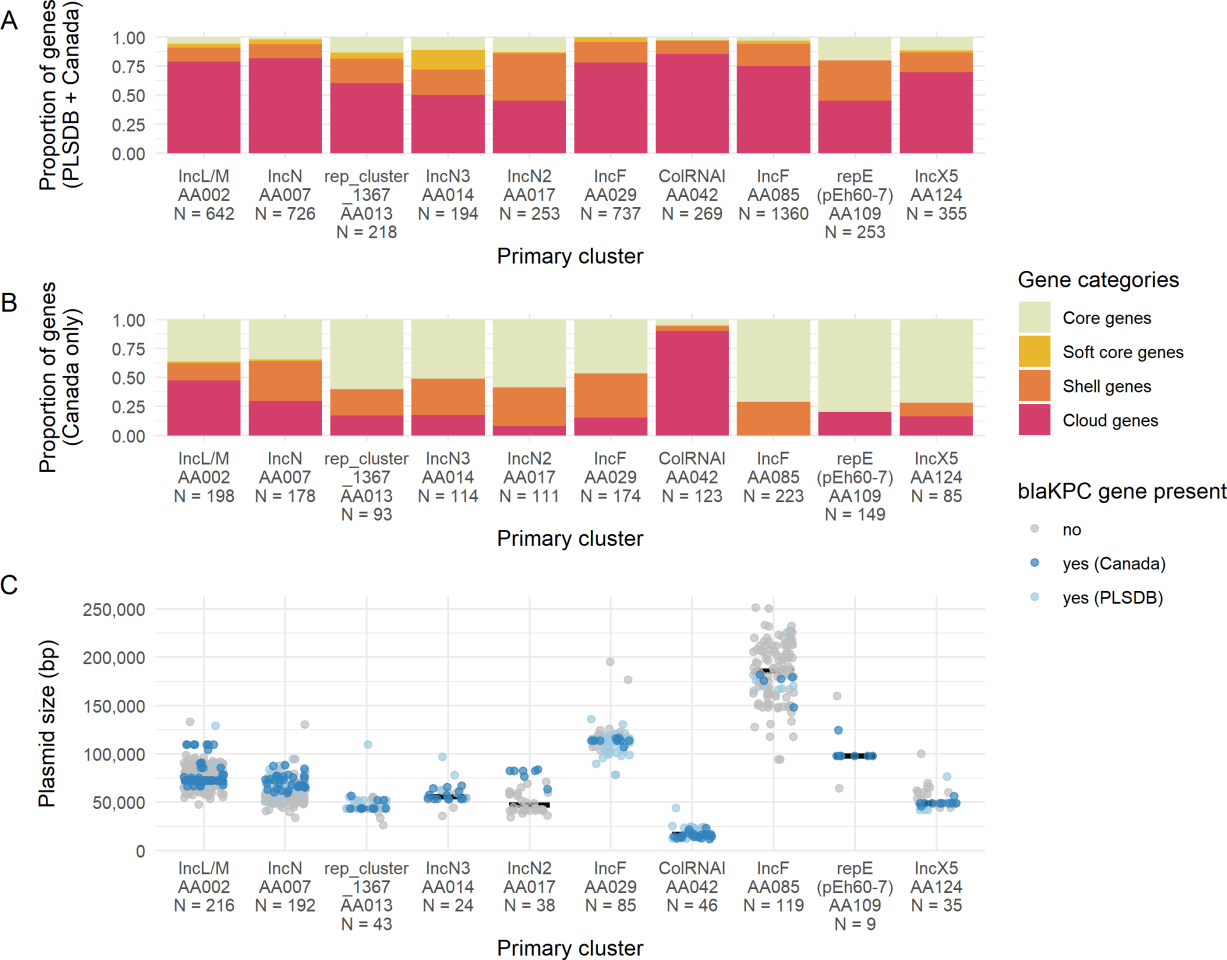
Pangenome size and *bla*_KPC_ prevalence among top 10 MOB-suite primary clusters identified among PLSDB and Canadian *bla*_KPC_-encoding plasmids. Pangenome size was calculated for (A) Canadian and PLSDB plasmids, and (B) Canadian plasmids only. Gene categories represent genes found in 99%–100% of plasmids (core), 95%–99% of plasmids (soft core), 15%–95% of plasmids (shell), and 0%–15% of plasmids (cloud). (C) Plasmid length in base pairs and prevalence of *bla*_KPC_ among plasmids in each primary cluster.

From the above data, we observed 10 primary clusters which represented 87% (175/202) of the Canadian *bla*_KPC_ plasmids from this study. The primary clusters generally reflected incompatibility groups and the major trends of each are summarized in Table 1. Within the global plasmid data set, the presence of *bla*_KPC_ varied between our top primary clusters (Fig. 4); some primary clusters tended to be frequently associated with *bla*_KPC_ (AA014, AA013, AA029, and AA109), whereas others appeared to encode *bla*_KPC_ more sporadically (AA085 and AA017). Some clusters had a tight distribution of plasmid sizes (AA013 and AA042) whereas others had a broader size distribution (AA085 and AA002). The percentage of core/soft core genes (defined as genes found in >95% plasmids) varied from 3% (AA042) to 28% (AA014) across the primary clusters, indicating some tend to have more diverse or dynamic gene content than others. We also evaluated the core genome size of the Canadian plasmids within each of these primary clusters as we suspected that the Canadian plasmids would be more closely related to each other than to global plasmids. As expected, the percentage of core genes within the Canadian plasmids was higher than the percentage predicted in the global data set with one exception (AA042) (Fig. 4). Several primary clusters had an average of five or more antimicrobial resistance genes (AA002, AA017, and AA085). Certain primary clusters had broad host ranges (AA002, AA007, and AA124), whereas others were only found in a single genus (AA085 and AA109). The dominant type of transposon was Tn*4401*b except for the IncF clusters (AA085 and AA029) where Tn*4401*a was dominant.

**TABLE 1:** Summary features of top 10 Canadian *bla*_KPC_-encoding plasmid clusters among PLSDB

Primary cluster ID	No. Canadian plasmids	Median size (kb)	Predicted mobility^*a*^^,*b*^	Replicon type^*a*^^,*b*^	Relaxase type^*a*^^,*b*^	MPF type^*a*^^,*b*^	No. genera	Core genes (%)^*d*^	*bla*_KPC_ allele^*a*^	*bla*_KPC_ frequency	Tn*4401* variant^*a*^	Mean no. ARGs per plasmid^*c*^	Plasmid taxonomic unit (PTU)^*e*^
AA002	62	75	Conjugative	IncL/M	MOBP	MPF_I	12	0.09	*bla*_KPC_-3	0.34	Tn*4401*b	5.1	L/M
AA007	31	57.3	Conjugative	IncN	MOBF	MPF_T	15	0.06	*bla*_KPC_-3	0.38	Tn*4401*b	6.1	N1
AA013	10	43.6	Mobilizable	Rep_cluster_1367	MOBC	−^*f*^	8	0.18	*bla*_KPC_-2	0.86	Tn*4401*b	2.5	E8
AA014	13	55.6	Conjugative	IncN	MOBF	MPF_T	4	0.28	*bla*_KPC_-3	0.71	Tn*4401*b	1.4	N2/3
AA017	7	47	Conjugative	IncN	MOBF	MPF_T	5	0.14	*bla*_KPC_-2	0.18	Tn*4401*b	5	N2/3
AA029	9	113.6	Conjugative	IncFIB, IncFII, rep_cluster_2183	MOBF, MOBF	MPF_F	4	0.04	*bla*_KPC_-2	0.86	Tn*4401*a	3.6	FK
AA042	21	16.5	Mobilizable	ColRNAI_rep_cluster_1857	MOBC	−	7	0.03	*bla*_KPC_-3	1	Tn*4401*b	1.7	Could not be assigned
AA085		185.7	Conjugative	IncFIB, IncFII, rep_cluster_2183	MOBF, MOBF	MPF_F	1	0.06	*bla*_KPC_-2	0.12	Tn*4401*a	5.9	FK
AA109	7	97.9	Non-mobilizable	Rep_cluster_2268 [novel repE(pEh60-7)]	−	MPF_F	1	0.2	*bla*_KPC_-3	0.78	Tn*4401*b	1	Could not be assigned
AA124	10	48.8	Conjugative	IncX5	MOBP	MPF_T	9	0.13	*bla*_KPC_-3	0.4	Tn*4401*b	1.6	New (putative) PTU

^*a*^ Values indicate the most common genotype in the cluster and may not apply to all plasmids in the cluster.

^*b*^ Values obtained from MOB-suite (24, 26). Mobility is assigned based on the presence of relaxase (mobilizable) and/or MPF proteins (conjugative) or absence of both (non-mobilizable).

^*c*^ ARGs = antimicrobial resistance genes.

^*d*^ Core genes represent the number of genes present in >95% of plasmids in the cluster, divided by the total number of non-redundant genes in the cluster.

^*e*^ PTU values obtained from COPLA (27) .

^*f*^ "-" indicates no relaxase or MPF protein detected.

Not surprisingly, all primary clusters with the exception of AA042 contain features that support their stability and persistence in the host cell. Of the genes found in >95% of plasmids in each cluster, between one and five genes (representing 2%–33% of genes per cluster) are involved in stability/transfer/defense which includes genes such as partition/stability genes (e.g., *parA*, *parM*, and *stbB*), anti-restriction genes (e.g., *ardA*, *ardB*, and *ardR*), and SOS-inhibition genes (e.g., *psiA* and *psiB*). Different primary clusters had different proportions of stability/transfer/defense gene content; some harbored a single anti-restriction protein (AA124), whereas some encoded two anti-restriction proteins, a plasmid stability protein, an endonuclease, and a stability protein (AA029).

### Canadian *bla*_KPC_-encoding plasmids in the top primary clusters

Given the high proportion of conserved genes among Canadian plasmids within each primary cluster (Fig. 4), we focused on the features of these Canadian plasmids separately from the other PLSDB plasmids present in each primary cluster. The primary clusters containing the most *bla*_KPC_-encoding Canadian plasmids were IncL/M replicons (AA002) and IncN replicons (AA007, AA014, and AA017) (Table 1) and are examined in more detail below.

**Fig 5:**
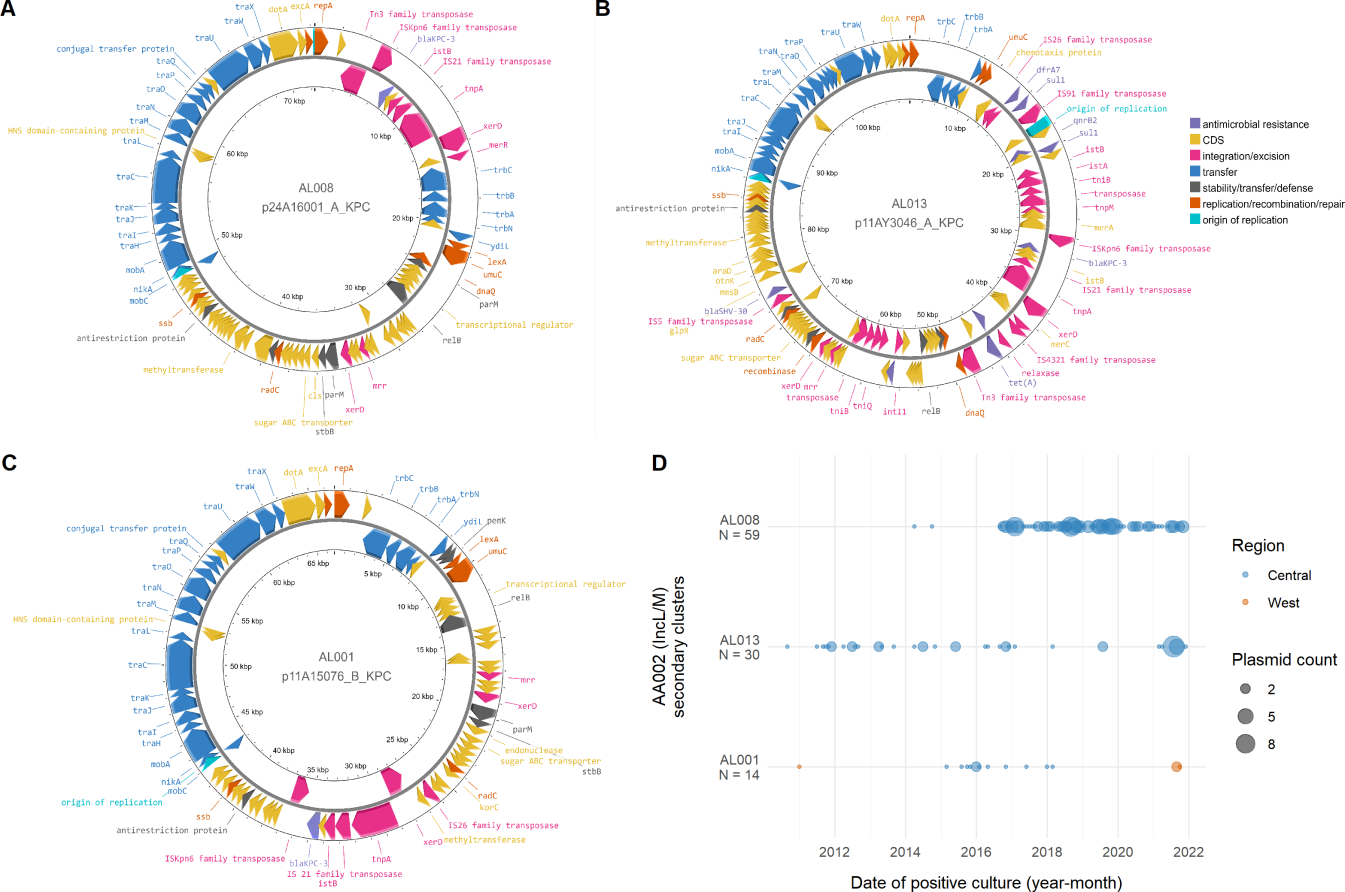
Diversity of secondary clusters within primary cluster AA002 (IncL/M). Plasmid structures of (A) secondary cluster AL008, (B) secondary cluster AL013, and (C) secondary cluster AL001 which grouped within the AA002 (IncL/M) primary cluster. Coding sequences are colored by function, and not all coding sequences are labeled. (D) Date of positive culture and region of plasmids from primary cluster AA002 (IncL/M). Date of positive culture was grouped into year-month bins. The Central region represents the Canadian provinces of Ontario and Québec, and the West region represents British Columbia, Alberta, Saskatchewan, and Manitoba. “N” indicates the number of isolates that contained assembled or predicted plasmids in the respective secondary clusters.

The Canadian plasmids in primary cluster AA002 (IncL/M) were classified into three secondary clusters: AL008 (49/62, 79%), AL013 (8/62, 13%), and AL001 (1/62, 1.6%) (Fig. 5). The first secondary cluster (AL008) had >99% nucleotide identity. They were 72.3 kb isolated from *K. pneumoniae* species complex, *K. aerogenes*, *K. oxytoca*, *C. freundii* complex, *E. cloacae* complex, *E. coli*, and *Raoultella planticola* between 2016 and 2020 from three sites in the same province. These plasmids harbor *bla*_KPC-3_ on Tn*4401*b-3 and do not encode any other resistance genes. The next secondary cluster (AL013) was also shared among multiple genera with >99% nucleotide identity. They were 109.8 kb isolated from *E. coli*, *K. pneumoniae* species complex, and *E. cloacae* complex from one site between 2011 and 2021. In addition to *bla*_KPC-3_ on Tn*4401*b-3, this plasmid also encodes *ant*(2″)-Ia, *bla*_SHV-30_, *dfrA7*, *qacE*, *qnrB2*, *tetA*, and two copies of *sul1*. Structurally, the secondary clusters have similar backbones of transfer and replication genes (Fig. 5). AL008 and AL013 have Tn*4401*b-3 inserted on the same strand, but AL013 contains many additional integration/excision mobile elements, antimicrobial resistance genes, and hypothetical coding sequences. The AL001 plasmid is structurally similar to AL008 plasmids, but has a different locus of insertion of Tn*4401*b-3 on the opposite strand and has a *pemKI* toxin-antitoxin system. The prevalence of these plasmids among different genera and the presence of conjugation genes (MOBP relaxase and MPF_I mating pair formation protein) suggests that regional horizontal transmission has contributed to these secondary clusters’ persistence.

Three primary clusters (AA007, AA014, and AA017) were identified as having IncN-type replicons with similar features (Table 1). Each cluster corresponds to a different type of replicon: IncN (AA007), IncN2 (AA017), and IncN3 (AA014). All plasmids in primary cluster AA007 were grouped in a single secondary cluster (31/31, 100%), as did all plasmids in primary cluster AA017 (7/7, 100%); in contrast, plasmids in primary cluster AA014 were split into two secondary clusters: AL059 (11/13, 84%) and AL060 (2/13, 15%). The first secondary cluster (AL059) was isolated from *E. coli*, *K. pneumoniae*, and *E. cloacae* complex from five sites in one province between 2014 and 2020. These plasmids encode *bla*_KPC-3_ on Tn*4401*b-2 and no other resistance genes with the exception of a single plasmid with a multi-resistance gene island. The other secondary cluster (AL060) was found in *C. freundii* complex and *K. pneumoniae* complex at two sites in one province. This group has no resistance genes aside from *bla*_KPC-2_ on Tn*4401*a-2. Primary cluster AA017 plasmids were isolated from *E. coli*, *K. pneumoniae* complex, *E. cloacae* complex, and *Citrobacter farmeri* from two sites in one province between 2016 and 2020. Aside from *bla*_KPC-2_ on Tn*4401*b-1, other resistance genes found in this cluster include *bla*_TEM-1B_, *dfrA25*, *mph(A)*, *qacE*, *qnrB2*, and *sul1*. Plasmids in the largest IncN primary cluster (AA007) were isolated from *E. coli*, *K. pneumoniae* complex, *Klebsiella oxytoca*, *Klebsiella michiganensis*, *Raoultella ornithinolytica*, *E. cloacae* complex, *C. freundii* complex, *Citrobacter amalonaticus*, *Citrobacter koseri*, and *Serratia marcescens*. These plasmids were isolated from 2012 to 2020 from nine sites in four provinces. This group contains *bla*_KPC-3_ on Tn*4401*b with many of them encoding different combinations of *aac*(6′)-Ib, *aph*(3″)-Ib, *aadA1*, *bla*_OXA-9_, *bla*_TEM-1A_, *dfrA14*, *qacE*, *qnrS1*, *sul1*, *sul2*, and *tetD*. The prevalence of IncN plasmids in multiple genera from multiple sites in the same province (primary clusters AA017 and AA014) or across multiple provinces (AA007) along with encoded conjugation genes (MOBF relaxase and MPF_T mating pair formation protein) suggests there has been widespread horizontal transmission of these primary clusters in Canada.

### Canadian *bla*_KPC_-encoding plasmids in the other primary clusters

Plasmids in primary cluster AA042 are small in size (10–20 kb) and grouped into four secondary clusters: AL143 (2/21, 10%), AL144 (17/21, 81%), AL149 (1/21, 5%), and AL150 (1/21, 5%). The largest secondary cluster (AL144) had multiple replicon types identified by MOB-suite, with the most common being ColRNAI_rep_cluster_1987 and rep_cluster_2335. These plasmids were isolated from 14 sites across three provinces from *C. freundii* complex, *E. coli*, *K. pneumoniae* complex, and *S. marcescens* between 2011 and 2021. These plasmids did not contain other resistance genes and most (14/17, 82%) were classified as “mobilizable” by MOB-suite, which indicates they encoded a relaxase or recognizable *oriT* sequence. The core genes in this group include all components of the Tn*4401*b transposon including *bla*_KPC-3_ which results in integration/excision genes being common in this secondary cluster. The small size of mobilizable plasmids in this primary cluster and their prevalence at various sites indicate that *bla*_KPC_ spread may be influenced by these smaller vectors.

**Fig 6:**
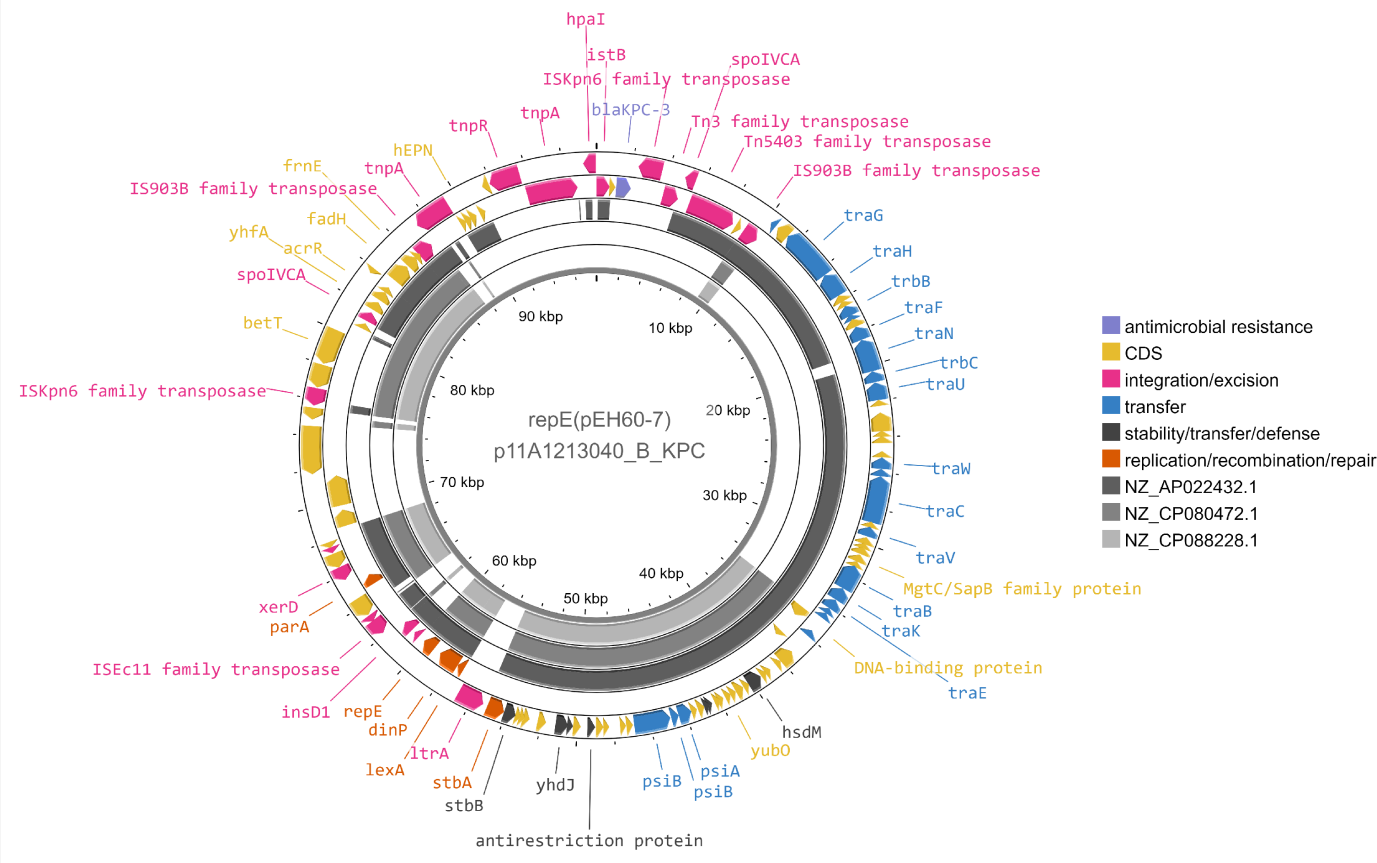
Comparison of p11A1314040_B_KPC, representative of plasmids with novel replicon repE(pEh60-7) and reference NZ_CP088228.1 (pEh60-7), NZ_CP080472.1 (PLSDB), and NZ_AP022432.1 (PLSDB) from cluster AA109. Reference sequences were aligned using blastn+ and an *e*-value cut-off of 0.1. NZ_CP080472.1 and NZ_AP022432.1 were placed in the same primary cluster by MOB-cluster as the reference p11A1314040_B_KPC but in different secondary clusters. Genes are colored by functional classes determined by mobileOG-db, and genes without annotations are hypothetical proteins. Not all gene labels are displayed.

We detected a novel replicon type in primary cluster AA109, designated as rep_cluster_2268 by MOB-typer, which now has a novel replicon designation of repE(pEh60-7) in PlasmidFinder (personal communication). We obtained complete plasmid sequences for seven isolates which grouped into a single secondary cluster. These plasmids were only found in the *Enterobacter cloacae* species complex, specifically *Enterobacter hormaechei* subsp. *hoffmannii* ST316, at a single site in one province from 2011 to 2020, indicating a very stable plasmid or reservoir of this strain. Occurrences of this plasmid are intermittent from 2011 to 2016 and appear to become more prevalent from 2016 to 2018 with fewer detected in recent years. SNVPhyl (28) was run to analyze SNV differences in the ST316 *bla*_KPC_-harboring isolates from this site over the 8-year time span (*n* = 51), and a maximum of 62 SNVs was identified between isolates using 88.51% of the genome. The novel repE(pEh60-7) plasmids encode *bla*_KPC-3_ in Tn*4401*b-3 but did not encode any other resistance genes. While they were classified as non-mobilizable by MOB-suite, they do encode a mating pair formation protein and other genes annotated as conjugation machinery which may not be represented in current databases (Fig. 6). Further work will need to be done to determine if these plasmids are conjugative. These plasmids also encode for stability/transfer/defense proteins including a SOS-inhibition protein, stability partition proteins, the *hok-sok* toxin-antitoxin system, and an anti-restriction protein. There were two PLSDB plasmids that grouped in the same primary AA109 cluster but in different secondary clusters, and their gene content is quite diverse compared to the Canadian plasmids (70% identity to NZ_AP022432.1 and 43% identity to NZ_CP080472.1) (Fig. 6). Given these plasmids are only found in one specific sequence type, clonal transmission is likely driving this primary cluster’s persistence.

Primary cluster AA124 plasmids were classified as IncX5 replicons grouped into two secondary clusters: AL404 (1/10, 10%) and AL405 (9/10, 10%). The largest secondary cluster (AL405) encoded *bla*_KPC-3_ on Tn*4401*b-3. These plasmids were isolated from *K. pneumoniae* complex, *Serratia liquefaciens*, *E. cloacae* complex, and *Klebsiella ascorbata* from four sites in two provinces between 2015 and 2021. Eight of these nine plasmids are >99% identical and do not contain any resistance genes aside from *bla*_KPC-3_ on Tn*4401*b-3. These plasmids are predicted to be conjugative based on the presence of the MOBP relaxase and MPF_T mating pair formation protein. The wide range of genera suggests that these IncX5 plasmids have disseminated horizontally across the country.

Two primary clusters (AA029 and AA085) contained IncF-type replicons that were found exclusively in *K. pneumoniae* isolates. All plasmids encoded two MOBF relaxases and an MPF_F mating pair formation protein which suggests that they are conjugative. Plasmids in primary cluster AA085 were grouped into a single secondary cluster and were isolated from two sites in two provinces from 2011 to 2020. These plasmids were from ST512 (3/5, 60%) and ST258 (2/5, 40%) isolates and varied in size (range: 148–182 kb). All plasmids contained Tn*4401*a, but *bla*_KPC-2_ was present in ST258 and *bla*_KPC-3_ was present in ST512, and all but one encoded *aadA2*, *aph*(3′)-Ia, *drfA12*, *mphA*, *qacE*, and *sul1*. Primary cluster AA029 plasmids were grouped into three secondary clusters which reflected the sequence type of the host: AL107 (ST512, 6/9, 67%), AL104 (ST258, 2/9, 22%), or AL106 (ST152, 1/9, 11%). Two of these secondary clusters (AL107, ST512; AL106, ST152) encoded *bla*_KPC-3_ on Tn*4401*a-7, whereas the other (AL104, ST258) encoded *bla*_KPC-2_ on Tn*4401*a-1. The secondary clusters AL107 and AL106 (ST512, ST152) were isolated from the same single site from 2010 to 2011, and the ST258 plasmids were isolated from two sites in one province in 2016 and 2019. Both secondary clusters AL107 and AL106 (ST512, ST152) also encoded *bla*_OXA-9_ and *bla*_TEM-1A_. One of the first described pKpQIL plasmids in *K. pneumoniae* ST258 [NC_014016.1 (29)] in PLSDB clustered within AA029 (secondary cluster AL107) and also encoded *bla*_OXA-9_ and *bla*_TEM-1A_. These pKpQIL-type plasmids have been globally disseminated through the expansion of *K. pneumoniae* clonal complex 258 (9, 29–31), and our results support continued clonal dissemination of these plasmid types in Canada.

### Epidemiology of *bla*_KPC_-encoding plasmid clusters across Canada

Long-read data are essential for resolving plasmid structures (32), but this can be costly for large surveillance data sets when performed alongside short-read sequencing, which then makes it difficult to analyze plasmid populations at a broad scale. Consequently, we explored MOB-recon (24) as a tool to predict plasmid cluster presence in isolates with incomplete *bla*_KPC_-encoding contigs (635/829, 76.6%) using a database containing a subset (~20%) of our isolates that were long-read sequenced. We successfully assigned the majority of *bla*_KPC_-encoding contigs to an existing plasmid primary cluster that we defined above, demonstrating that MOB-recon is a feasible approach to predict plasmid cluster membership without long-read sequencing all isolates.

MOB-recon assigned 95% (603/635) of incomplete *bla*_KPC_-encoding contigs to an existing primary cluster, despite approximately half of *bla*_KPC_ contigs (302/635, 48%) not matching any replicon, relaxase, MPF, or *oriT* sequence in the MOB-suite database. The 10 largest primary clusters contained 89% (539/603) of *bla*_KPC_ contigs. Similar to the trends observed above (Table 1), the largest number of *bla*_KPC_ contigs were classified into the IncL/M cluster (AA002, 142/635, 22%) and the IncN clusters (AA007, 149/635, 23%; AA014, 23/635, 3.6%; and AA017, 27/635, 4.3%) (Fig. S1). All contigs assigned to primary cluster repE(pEh60-7) (AA109) (34/635 contigs, 5%) belonged to *E. hormaechei* subsp. *hoffmannii* ST316, and all contigs assigned to the IncF primary cluster (representing both AA029 and AA085) (94/635, 15%) were from the *K. pneumoniae* species complex, which further confirms that these two primary clusters are species-specific. Finally, the remaining contigs were placed in the ColRNAI cluster (AA042, 27/635, 4%) the IncX5 cluster (AA124, 16/635, 3%), and the rep_cluster_1367 (AA109, 27/635, 5%). This reflects the distribution of complete *bla*_KPC_ plasmid clustering alongside PLSDB as described above (Fig. 4; Table 1). Several other replicon types of *bla*_KPC_-encoding contigs were present at low frequencies (21 IncHI2A, 14 IncX3, 12 IncC, and 9 IncP). Those contigs that were unclassified appeared to be chromosomal (located on contigs >1 Mb; 3/32, 9%), coincided with the 10 kb length of Tn*4401* and were filtered out (6/32, 19%), or did not match any plasmids currently in the database, indicating that further sequencing is required to confirm the genomic location of the *bla*_KPC_ in these isolates.

**Fig 7:**
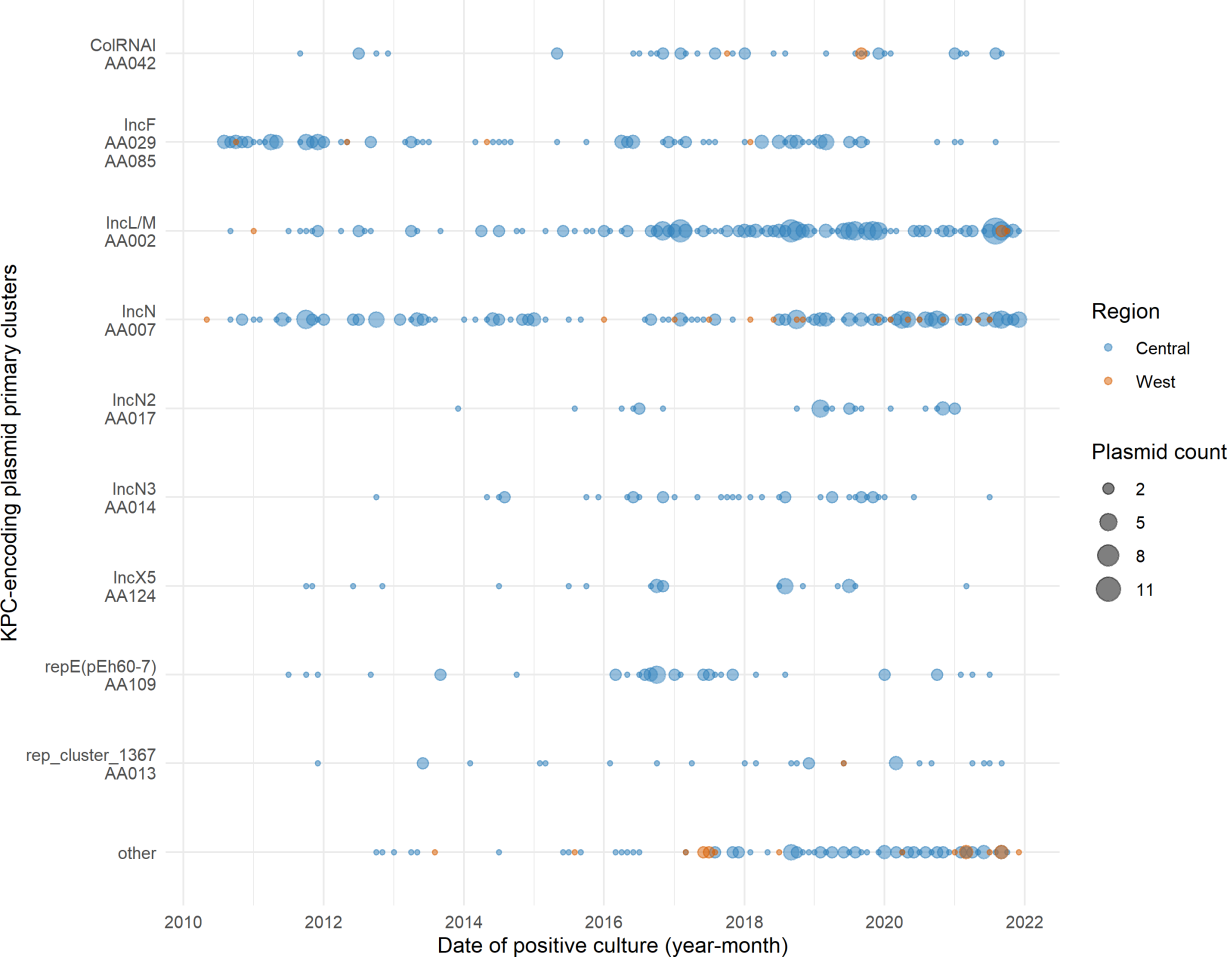
Epidemiology of Canadian *bla*_KPC_-encoding plasmid primary clusters from 2011 to 2021. Date of positive culture was grouped into year-month bins. The Central region represents the Canadian provinces of Ontario and Québec, and the West region represents British Columbia, Alberta, Saskatchewan, and Manitoba. “Other” indicates plasmids which did not group in the top 10 primary clusters.

Given the majority of *bla*_KPC_-encoding contigs were predicted to be of plasmid origin, we examined the temporal and geographic patterns of all *bla*_KPC_-encoding plasmids in Canada from 2010 to 2021 (Fig. 7). Geographically, the incidence of *bla*_KPC_-encoding isolates is much higher in the Central region than the West (Central: *n* = 777/829, 93.7%; West: *n* = 52/829, 6.3%). The three primary clusters that have persisted over the longest time frame are IncN (AA007), AA002 (IncL/M), and AA0029/AA085 (IncF). Occurrences of AA002 (IncL/M) plasmids were low until 2016, but appear to be found more frequently for the remainder of the study period. Some of the earliest detected plasmids were AA029/AA085 (IncF) plasmids, but these were observed less frequently from 2020 onward, perhaps as AA002 (IncL/M) plasmids increased in prevalence in the Central region. The AA007 (IncN) plasmids appear to be established in the population. Since 2018, there has been an increase in replicons in the “Other” category or those plasmids that do not fall into the top 10 primary clusters, particularly in the West region. This may indicate that other plasmids are becoming dominant in the West region, and further investigation into smaller Western-specific clusters (such as plasmids with IncP6 replicons) is required.

We examined the epidemiology of the three secondary clusters in primary cluster AA002 (IncL/M) in more detail, as this is the only primary cluster wherein we had a substantial number of plasmids in multiple secondary clusters (Fig. 5D). From an epidemiological perspective, AL013 plasmids have been present for a longer time frame at low abundances with an increase in prevalence in 2021, whereas AL008 plasmids have appeared consistently since 2016. It appears that secondary cluster AL001 may be spreading to the Western region, as this replicon was detected initially in the West in 2010 but not again until 2021 (Fig. 5D). The independent samples collected over long time periods indicate that these plasmids seem to be stably circulating in these regions.

## DISCUSSION

We examined the prevalence and distribution of *bla*_KPC_-producing *Enterobacterales* and their plasmids in Canada from 2010 to 2021. Our results suggest that a combination of regional clonal transmission (AA109, AA029, and AA085), regional horizontal transmission (AA017, AA124, AA002, and AA014), widespread horizontal transmission (AA007), and other means of mobilization (AA013 and AA042) has contributed to *bla*_KPC_ prevalence in Canada.

Here, we report on a novel *bla*_KPC_ plasmid, repE(pEh60-7), isolated exclusively from *E. hormaechei* subsp. *hoffmannii* ST316, which has been stably circulating in this population for over 8 years. There have been limited reports on the *E. cloacae* complex ST316 lineage elsewhere; one study identified nine *E. hormaechei* ST316 isolates encoding *bla*_KPC-2_ in the United Kingdom between 2014 and 2016 as part of a larger reference study, however, the plasmid content was not reported (33). Otherwise, eight isolates [one each from references (34–36), and five from reference (37)] of *E. hormaechei* ST316 have been recently been reported to carry *bla*_NDM-1_ in Asia. Although conjugation genes were present, the narrow host range of the plasmid suggests that *bla*_KPC_ persistence is likely due to the ST316 clone and not driven by the plasmid.

We report on some plasmid clusters that are found across a variety of species, supporting evidence of horizontal transfer of *bla*_KPC_. One such primary cluster was AA002, which contained the IncL/M plasmids isolated from *Citrobacter* spp., *Escherichia* spp., *Enterobacter* spp., *Klebsiella* spp., and *Raoultella* spp. isolates. The secondary cluster AL008 plasmids had a single resistance gene, but secondary cluster AL013 plasmids encoded fluoroquinolone resistance genes, additional β-lactamase genes, and other antimicrobial resistance genes which may favor their persistence in hospital environments. In addition, all IncL/M plasmids encoded a ParB-like partitioning/stability protein, an anti-restriction protein, a restriction endonuclease (Mrr), single-stranded DNA-binding protein (Ssb), and the RelB antitoxin although no RelE toxin was detected in any plasmid sequence. These features likely contribute to their long-term persistence and stability in Canada. However, *bla*_KPC_-encoding IncL/M replicons appear to be rare as they are notably absent from other recent *bla*_KPC_ surveillance studies (13, 15, 38, 39). One study reported that *bla*_KPC-2_ being associated with IncL/M replicons a multi-clonal *K. pneumoniae* outbreak in a region in Argentina, but the relatedness between plasmids was not investigated (40). IncL/M plasmids are typically associated with *bla*_NDM_, *bla*_IMP_, and *bla*_OXA_-type carbapenemases (3, 12, 13, 41–43), although a select few cases have been reported in diverse hosts (19, 44–47). The pNE1280 IncL/M plasmid, which was one of the first IncL/M plasmids described carrying *bla*_KPC-2_ (45), clustered in the AL001 secondary cluster, which was not well represented in the Canadian data set. We demonstrated ongoing *bla*_KPC_-encoding IncL/M plasmid transfer across multiple genera which merits further investigation of IncL/M-type replicons as potential vectors for long-term *bla*_KPC_ persistence.

In contrast to the IncL/M plasmids, the IncN plasmid family had the broadest phylogenetic and geographical distribution across Canada, which is unsurprising given their broad host ranges and their long association with *bla*_KPC_ genes (3, 48, 49). *bla*_KPC_-encoding IncN plasmids have been linked to multi-center outbreaks in Columbia (50) and Germany (51), both facilitated by intra- and inter-species horizontal plasmid transfer. Other *bla*_KPC_-encoding IncN plasmids implicated in inter-patient transfer grouped in the same secondary clusters as the Canadian plasmids here [plasmid 12 (49) and pYDC107_70 (52) in AA007/AL033; pKPC-SMH (53) in AA014/AL059], indicating high sequence similarity among Canadian plasmids to those found across the globe. Similar to IncL/M plasmids, all IncN plasmids encoded partitioning/stability proteins (StbA, StbC, and StdB), at least two anti-restriction proteins (ArdA, ArdB, and ArdR), an endonuclease (EcoRII and PemK), and other antimicrobial resistance genes. Given these features are also common in IncL/M plasmids, further investigation is required to explain why the IncN plasmids have a broader geographic and host range distribution in Canada than the IncL/M plasmids.

The IncF-type replicons observed here [IncFIB(pQil)/IncFII(K) and IncFIB(K)/IncFII(K)] are common in *bla*_KPC_-producing *K. pneumoniae* species complex isolates detected in other nationwide surveillance programs (12, 13, 15, 17, 18, 38, 39, 54). IncF-type replicons have been implicated in clinical outbreaks of *bla*_KPC_-producing *K. pneumoniae* in the Netherlands (55) and the United States (56) as well as cross-species plasmid transmission in the United Kingdom (57). The clonal lineage ST258/512 accounts for 39.6% (106/268) of *bla*_KPC_-producing *K. pneumoniae* isolates in our collection (Fig. 1) which were isolated across 3 provinces and 12 sites. This frequency is lower than frequencies observed in Colombia [47% (18)], in the United States [74% CRACKLE-2 (5)], and across Europe [73% EuSCAPE (13), 76% Europe/Israel (39)], but is higher than frequencies reported in the United Kingdom [20% (15)] and Argentina [0% (40)]. Our findings support that IncF-type plasmids in *K. pneumoniae* ST512 and ST258 are important in Canada for *bla*_KPC_ persistence in the clonal group ST258/512, although there is more diversity in the sequence type of *bla*_KPC_-encoding *K. pneumoniae* isolates in Canada compared to other parts of the world. Interestingly, IncF-type plasmids were less common in recent years (2020 and 2021), indicating other plasmid types such as IncL/M and IncN-type replicons may be replacing them in the Canadian landscape.

Small cryptic plasmids are often overlooked (58) and underrepresented in plasmid-mediated antimicrobial resistance studies (59). ColRNA-type replicons smaller than 22 kb were the third largest *bla*_KPC_-encoding primary clusters in our data set. ColRNA-type plasmids were predicted to be found in >90% of carbapenemase-producing *Klebsiella pneumoniae* isolates across Europe (13, 60) and around the world (61), although these authors did not investigate if any carbapenemase genes were found on these plasmids. Similarly, ColRNAI replicons were present in >50% of *bla*_KPC_-producing isolates in Colombia (18). Our MOB-recon results agree with these data and predict that 69% (568/829) of Canadian isolates contain ColRNAI-type or rep_cluster_2335 plasmids, the majority (524/568, 92%) of which do not encode *bla*_KPC_ (data not shown). These plasmids can be important vectors for transferring antimicrobial resistance genes between isolates, including the transfer of *bla*_KPC_-type carbapenemases (19, 31, 62). Experimentally, ColRNAI plasmids encoding *bla*_KPC_ and Tn*4401* could mobilize between strains and participate in Tn*4401* transposition events within a patient (63). About half the plasmids we sequenced in this primary cluster do not encode a relaxase but they could be mobilized by the relaxase of a co-resident plasmid if the *oriT* sequence has enough similarity as shown in a previous study (64). Given their high prevalence and ability to mobilize resistance elements, these small plasmids are likely contributing to *bla*_KPC_ dissemination and merit further investigation as potential vectors for *bla*_KPC_ and other antimicrobial resistance spread across the globe.

Plasmid clustering and analyses require a methodology that is tolerant to a small amount of changes given the plasticity of plasmid sequences. Clustering plasmids with MOB-cluster provided a way to analyze subsets of similar plasmids, and MOB-recon allowed us to predict the prevalence of these plasmid clusters in isolates with incomplete assemblies. Canadian plasmids are grouped in two or three secondary clusters within each primary cluster (Mash distance <0.025), with typically a single secondary cluster containing the majority of Canadian plasmids in that primary cluster, indicating many of them have near duplicate sequences (26, 65) and this provided a starting point for investigating clusters of interest in more detail.

Analyzing broad-scale plasmid populations is desirable for surveillance programs, and ideally, every isolate would be sequenced to obtain complete plasmid sequences. Short-read sequencing alone results in many plasmids remaining fragmented (32) but performing long-read sequencing in addition to short-read on large collections of isolates can be cost and time-prohibitive, so we explored using MOB-recon to predict putative plasmids in incomplete assemblies after long-read sequencing a subset of isolates. There is no standardized method for estimating plasmid presence/containment in fragmented assemblies. It is important to recognize that any reference-based method for plasmid reconstruction involving mapping to reference sequences to infer presence assumes that plasmid structures are relatively conserved, and this approach can produce misleading results if plasmid plasticity is high (66, 67). Similar studies have used short-read mappers such as BWA/Bowtie2/SMALT (13, 43, 68, 69) or k-mer-based tools (60, 70) to determine plasmid prevalence in incomplete assemblies, typically using a select few plasmids as references. We use a homology-based clustering approach with MOB-recon (24) to reconstruct plasmids using a relatively large set of complete Canadian plasmids (1,856 plasmids). Previous work has shown that MOB-recon performed well compared to other tools (58, 71, 72), and that *Enterobacterales* plasmid replicons and mobility genes are well represented in the MOB-suite database (58). Using this approach, we were able to predict plasmids for the majority of incomplete *bla*_KPC_-encoding contigs (605/635, 95%). For several primary clusters that were only present in specific sequence types [IncF-type, AA029/AA085 and repE(pEh60-7), AA109], those *bla*_KPC_-encoding contigs that were predicted to be part of plasmids in those primary clusters were from isolates with matching sequence types, which further supports this methodology for plasmid analysis in large-scale *Enterobacterales* surveillance data and eliminates the requirement to perform long-read sequencing on every single isolate.

In summary, there are multiple distinct clusters of *bla*_KPC_-encoding plasmids that circulated in Canada from 2010 to 2021. Certain plasmid clusters spread by horizontal transmission and were found in multiple genera in multiple provinces whereas others persisted through clonal dissemination of the host organism. Our findings highlight the need to integrate targeted long-read sequencing into carbapenemase-producing organism surveillance to generate complete plasmid assemblies and demonstrate that plasmid clustering can facilitate analyses of a large number of plasmids. The characterization of *bla*_KPC_ plasmids is not only important for outbreak control but also for epidemiological surveillance of antimicrobial resistance, as plasmids encoding *bla*_KPC_ genes typically carry other antimicrobial resistance genes that can be exchanged between species.

## MATERIALS AND METHODS

### Surveillance period and PCR confirmation of *bla*_KPC_ carbapenemase gene

CNISP is a sentinel surveillance system which collects epidemiological and linked microbiology data from 90 Canadian acute-care hospitals across 10 provinces and 2 territories. *Enterobacterales* isolates non-susceptible to a carbapenem (as determined by the submitting site) isolated from patients from 2010 to 2021 were voluntarily submitted to the National Microbiology Laboratory (Winnipeg, Canada) by Canadian hospitals and provincial public health laboratories for carbapenemase gene detection. Multiplex PCR to confirm the carbapenemase gene *bla*_KPC_ was conducted as previously described (54). A total of 829 isolates encoding *bla*_KPC_ were collected from 2010 to 2021 from 34 hospitals (Table S1), with one hospital submitting 38% (317/829) of all isolates. Where applicable, the Central region refers to the provinces of Ontario and Québec, and the West region refers to the provinces of British Columbia, Alberta, Saskatchewan, and Manitoba. See the Supplemental Materials for additional information about the surveillance program and isolate eligibility criteria.

### Whole genome sequencing

All 829 isolates were sequenced with Illumina MiSeq platforms, and 155 of these were additionally sequenced using Oxford Nanopore Technologies (ONT). Isolates for ONT long-read sequencing represented about 20% of all *bla*_KPC_ cases stratified across all provinces in Canada. For sites with known outbreaks, we took representative isolates to prevent oversampling of clonal cases. The average Illumina depth of coverage was 123×, and the average ONT depth of coverage was 75×. Genomes were trimmed and filtered before assembly with Unicycler v0.5.0 (73). A total of 111 chromosomes and 1,856 plasmids were completed and circularized in our data set. Details are in the Supplementary Materials, and details on *bla*_KPC_-encoding plasmids can be found in Table S2.

### Bioinformatic analyses

Organism genus was confirmed using the RefSeq Masher Matches tool (74) and FastANI v1.3 (75) for *Enterobacter* spp. Species complex definitions are in the Supplementary Materials. StarAMR v0.9.1 (76) was used to detect antimicrobial resistance genes using the ResFinder database v2022-05-24 (77) and sequence type using the multi-locus species types database v2.23.0 (78, 79). TETyper v1.1 (23) was used to identify the Tn*4401* isoforms using the reference Tn*4401*b-1 found in the GitHub repository (accession CP017937.1:29609-39614) and variants using an updated SNP profile file (Table S3) and an updated structural profile file (Table S4) based on the literature (22, 23, 45, 80–84). Panaroo v1.3.2 (85) was used to estimate the pangenome and generate core gene alignments for each primary cluster as well as alignments for each of the top four genera (*Citrobacter*, *Enterobacter*, *Escherichia*, and *Klebsiella*). The MOB-typer tool from MOB-suite v3.1.4 (24, 26) was used to identify plasmid replicons and mobility classes using the default databases.

### Plasmid clustering and containment analysis

MOB-suite primary cluster designations are a useful way to broadly cluster plasmids for epidemiological studies, and so plasmids assigned to different primary MOB-clusters are sufficiently unrelated to not be considered as part of an epidemiologically relevant transmission event (26, 65). However, plasmids that share the same primary cluster designation can be examined in more detail through higher resolution subtyping such as secondary cluster designations. If two plasmids are assigned to the same secondary cluster, they have near duplicate sequences and are sufficiently related to be strong candidates for outbreak investigations (26, 65). In addition to secondary cluster designation, epidemiological data are required to best assess direct plasmid transmission.

For plasmid clustering analysis, the PLSDB v2021_06_23v2 (25) database was downloaded and clustered alongside the 202 circular *bla*_KPC_-encoding plasmids in this study using MOB-cluster from the MOB-suite v3.1.4 package (24, 26). For plasmid containment analysis, all 1,856 circular plasmids completed in this study (including the 202 *bla*_KPC_-encoding plasmids) were clustered using MOB-cluster to create a custom Canadian plasmid database. All 829 isolates were screened for plasmids with MOB-recon using this custom database, and the output was filtered to focus on the reconstructed plasmids containing *bla*_KPC_. Further details on plasmid clustering and containment are provided in the Supplementary Materials.

## Data Availability

Raw sequencing reads were deposited to the NCBI SRA archive under BioProject PRJNA855907. See Table S1 for a list of accessions. Complete *bla*_KPC_-encoding plasmid sequences were deposited to NCBI GenBank under the accessions listed in Table S2. Updated SNV profiles used for Tn*4401* typing with TETyper are found in Table S3 and updated structural profiles in are found in Table S4.

## SUPPLEMENTAL MATERIAL

Figure S1 aac.00860-23-s0001.tif Prediction of blaKPC-encoding plasmids from incomplete assemblies using MOB-recon.

Additional method details aac.00860-23-s0002.docx Additional method details.

Table S1 aac.00860-23-s0003.xlsx *bla*_KPC_-encoding isolate summary data.

Table S2 aac.00860-23-s0004.xlsx Complete *bla*_KPC_-encoding plasmid summary data.

Table S3 aac.00860-23-s0005.xlsx Updated Tn*4401* SNV table used for TETyper analysis.

Table S4 aac.00860-23-s0006.xlsx Updated Tn*4401* structural table used for TETyper analysis.

ASM does not own the copyrights to Supplemental Material that may be linked to, or accessed through, an article. The authors have granted ASM a non-exclusive, world-wide license to publish the Supplemental Material files. Please contact the corresponding author directly for reuse.

## References

[1] Sheu C-C, Chang Y-T, Lin S-Y, Chen Y-H, Hsueh P-R. 2019. Infections caused by carbapenem-resistant Enterobacteriaceae: an update on therapeutic options. Front Microbiol 10:80. doi:10.3389/fmicb.2019.00080 30761114 PMC6363665

[2] Queenan AM, Bush K. 2007. Carbapenemases: the versatile β-lactamases. Clin Microbiol Rev 20:440–458. doi:10.1128/CMR.00001-07 17630334 PMC1932750

[3] Kopotsa K, Osei Sekyere J, Mbelle NM. 2019. Plasmid evolution in carbapenemase-producing Enterobacteriaceae: a review. Ann N Y Acad Sci 1457:61–91. doi:10.1111/nyas.14223 31469443

[4] van Duin D, Doi Y. 2017. The global epidemiology of carbapenemase-producing Enterobacteriaceae. Virulence 8:460–469. doi:10.1080/21505594.2016.1222343 27593176 PMC5477705

[5] van Duin D, Arias CA, Komarow L, Chen L, Hanson BM, Weston G, Cober E, Garner OB, Jacob JT, Satlin MJ, et al.. 2020. Molecular and clinical epidemiology of carbapenem-resistant Enterobacteriaceae in the United States: a prospective cohort study. Lancet Infect Dis 20:731–741. doi:10.1016/S1473-3099(19)30755-8 32151332 PMC7473597

[6] Mataseje LF, Boyd DA, Fuller J, Haldane D, Hoang L, Lefebvre B, Melano RG, Poutanen S, Van Caeseele P, Mulvey MR. 2018. Characterization of OXA-48-like carbapenemase producers in Canada, 2011–14. J Antimicrob Chemother 73:626–633. doi:10.1093/jac/dkx462 29272439

[7] Canadian Nosocomial Infection Surveillance Program. 2023. Healthcare-associated infections and antimicrobial resistance in Canadian acute care hospitals, 2017–2021. Can Commun Dis Rep 49:235–252. doi:10.14745/ccdr.v49i05a09 PMC10903608 38425696

[8] Yigit H, Queenan AM, Anderson GJ, Domenech-Sanchez A, Biddle JW, Steward CD, Alberti S, Bush K, Tenover FC. 2001. Novel carbapenem-hydrolyzing β-lactamase, KPC-1, from a carbapenem-resistant strain of Klebsiella pneumoniae. Antimicrob Agents Chemother 45:1151–1161. doi:10.1128/AAC.45.4.1151-1161.2001 11257029 PMC90438

[9] Pitout JDD, Nordmann P, Poirel L. 2015. Carbapenemase-producing Klebsiella pneumoniae, a key pathogen set for global nosocomial dominance. Antimicrob Agents Chemother 59:5873–5884. doi:10.1128/AAC.01019-15 26169401 PMC4576115

[10] Nordmann P, Cuzon G, Naas T. 2009. The real threat of Klebsiella pneumoniae carbapenemase-producing bacteria. Lancet Infect Dis 9:228–236. doi:10.1016/S1473-3099(09)70054-4 19324295

[11] Munoz-Price LS, Poirel L, Bonomo RA, Schwaber MJ, Daikos GL, Cormican M, Cornaglia G, Garau J, Gniadkowski M, Hayden MK, Kumarasamy K, Livermore DM, Maya JJ, Nordmann P, Patel JB, Paterson DL, Pitout J, Villegas MV, Wang H, Woodford N, Quinn JP. 2013. Clinical epidemiology of the global expansion of Klebsiella pneumoniae carbapenemases. Lancet Infect Dis 13:785–796. doi:10.1016/S1473-3099(13)70190-7 23969216 PMC4673667

[12] Mathers AJ, Peirano G, Pitout JDD. 2015. The role of epidemic resistance plasmids and international high-risk clones in the spread of multidrug-resistant Enterobacteriaceae. Clin Microbiol Rev 28:565–591. doi:10.1128/CMR.00116-14 25926236 PMC4405625

[13] David S, Cohen V, Reuter S, Sheppard AE, Giani T, Parkhill J, European Survey of Carbapenemase-Producing *Enterobacteriaceae* (EuSCAPE) Working Group, ESCMID Study Group for Epidemiological Markers (ESGEM), Rossolini GM, Feil EJ, Grundmann H, Aanensen DM. 2020. Integrated chromosomal and plasmid sequence analyses reveal diverse modes of carbapenemase gene spread among Klebsiella pneumoniae. Proc Natl Acad Sci U S A 117:25043–25054. doi:10.1073/pnas.2003407117 32968015 PMC7587227

[14] Tofteland S, Naseer U, Lislevand JH, Sundsfjord A, Samuelsen O. 2013. A long-term low-frequency hospital outbreak of KPC-producing Klebsiella pneumoniae involving Intergenus plasmid diffusion and a persisting environmental reservoir. PLoS One 8:e59015. doi:10.1371/journal.pone.0059015 23536849 PMC3594221

[15] Davies TJ, Stoesser N, Sheppard AE, Abuoun M, Fowler P, Swann J, Quan TP, Griffiths D, Vaughan A, Morgan M, Phan HTT, Jeffery KJ, Andersson M, Ellington MJ, Ekelund O, Woodford N, Mathers AJ, Bonomo RA, Crook DW, Peto TEA, Anjum MF, Walker AS. 2020. Genomic epidemiology of complex, multispecies, plasmid-borne bla_KPC_ carbapenemase in Enterobacterales in the United Kingdom from 2009 to 2014. Antimicrob Agents Chemother 64:e02244–19. doi:10.1128/AAC.02026-19 32094139 PMC7179641

[16] Weingarten RA, Johnson RC, Conlan S, Ramsburg AM, Dekker JP, Lau AF, Khil P, Odom RT, Deming C, Park M, Thomas PJ, NISC Comparative Sequencing Program, Henderson DK, Palmore TN, Segre JA, Frank KM. 2018. Genomic analysis of hospital plumbing reveals diverse reservoir of bacterial plasmids conferring carbapenem resistance. mBio 9:e02011-17. doi:10.1128/mBio.02011-17 29437920 PMC5801463

[17] Shropshire WC, Dinh AQ, Earley M, Komarow L, Panesso D, Rydell K, Gómez-Villegas SI, Miao H, Hill C, Chen L, Patel R, Fries BC, Abbo L, Cober E, Revolinski S, Luterbach CL, Chambers H, Fowler VG, Bonomo RA, Shelburne SA, Kreiswirth BN, van Duin D, Hanson BM, Arias CA. 2022. Accessory genomes drive independent spread of carbapenem-resistant Klebsiella pneumoniae clonal groups 258 and 307 in Houston, TX. mBio 13:e0049722. doi:10.1128/mbio.00497-22 35357213 PMC9040855

[18] Saavedra SY, Bernal JF, Montilla-Escudero E, Arévalo SA, Prada DA, Valencia MF, Moreno J, Hidalgo AM, García-Vega ÁS, Abrudan M, Argimón S, Kekre M, Underwood A, Aanensen DM, Duarte C, Donado-Godoy P, NIHR Global Health Research Unit on Genomic Surveillance of Antimicrobial Resistance. 2021. Complexity of genomic epidemiology of carbapenem-resistant Klebsiella pneumoniae isolates in Colombia urges the reinforcement of whole genome sequencing-based surveillance programs. Clin Infect Dis 73:S290–S299. doi:10.1093/cid/ciab777 34850835 PMC8634422

[19] Stoesser N, Sheppard AE, Peirano G, Anson LW, Pankhurst L, Sebra R, Phan HTT, Kasarskis A, Mathers AJ, Peto TEA, Bradford P, Motyl MR, Walker AS, Crook DW, Pitout JD. 2017. Genomic epidemiology of global Klebsiella pneumoniae carbapenemase (KPC)-producing Escherichia coli. Sci Rep 7:5917. doi:10.1038/s41598-017-06256-2 28725045 PMC5517641

[20] Brandt C, Viehweger A, Singh A, Pletz MW, Wibberg D, Kalinowski J, Lerch S, Müller B, Makarewicz O. 2019. Assessing genetic diversity and similarity of 435 KPC-carrying plasmids. Sci Rep 9:11223. doi:10.1038/s41598-019-47758-5 31375735 PMC6677891

[21] Cuzon G, Naas T, Nordmann P. 2011. Functional characterization of Tn4401, a Tn3-based transposon involved in bla_KPC_ gene mobilization. Antimicrob Agents Chemother 55:5370–5373. doi:10.1128/AAC.05202-11 21844325 PMC3195030

[22] Naas T, Cuzon G, Villegas M-V, Lartigue M-F, Quinn JP, Nordmann P. 2008. Genetic structures at the origin of acquisition of the β-lactamase bla_KPC_ gene. Antimicrob Agents Chemother 52:1257–1263. doi:10.1128/AAC.01451-07 18227185 PMC2292522

[23] Sheppard AE, Stoesser N, German-Mesner I, Vegesana K, Sarah Walker A, Crook DW, Mathers AJ. 2018. TETyper: a bioinformatic pipeline for classifying variation and genetic contexts of transposable elements from short-read whole-genome sequencing data. Microb Genom 4:e000232. doi:10.1099/mgen.0.000232 30465646 PMC6412039

[24] Robertson J, Nash JHE. 2018. MOB-suite: software tools for clustering, reconstruction and typing of plasmids from draft assemblies. Microb Genom 4:e000206. doi:10.1099/mgen.0.000206 30052170 PMC6159552

[25] Schmartz GP, Hartung A, Hirsch P, Kern F, Fehlmann T, Müller R, Keller A. 2022. PLSDB: advancing a comprehensive database of bacterial plasmids. Nucleic Acids Res 50:D273–D278. doi:10.1093/nar/gkab1111 34850116 PMC8728149

[26] Robertson J, Bessonov K, Schonfeld J, Nash JHE. 2020. Universal whole-sequence-based plasmid typing and its utility to prediction of host range and epidemiological surveillance. Microb Genom 6:mgen000435. doi:10.1099/mgen.0.000435 32969786 PMC7660255

[27] Redondo-Salvo S, Bartomeus-Peñalver R, Vielva L, Tagg KA, Webb HE, Fernández-López R, de la Cruz F. 2021. COPLA, a Taxonomic Classifier of Plasmids. BMC Bioinformatics 22:390. doi:10.1186/s12859-021-04299-x 34332528 PMC8325299

[28] Petkau A, Mabon P, Sieffert C, Knox NC, Cabral J, Iskander M, Iskander M, Weedmark K, Zaheer R, Katz LS, Nadon C, Reimer A, Taboada E, Beiko RG, Hsiao W, Brinkman F, Graham M, Van Domselaar G. 2017. SNVPhyl: a single nucleotide variant phylogenomics pipeline for microbial genomic epidemiology. Microb Genom 3:e000116. doi:10.1099/mgen.0.000116 29026651 PMC5628696

[29] Leavitt A, Chmelnitsky I, Carmeli Y, Navon-Venezia S. 2010. Complete nucleotide sequence of KPC-3-encoding plasmid pKpQIL in the epidemic Klebsiella pneumoniae sequence type 258. Antimicrob Agents Chemother 54:4493–4496. doi:10.1128/AAC.00175-10 20696875 PMC2944570

[30] Chen L, Chavda KD, Melano RG, Jacobs MR, Koll B, Hong T, Rojtman AD, Levi MH, Bonomo RA, Kreiswirth BN. 2014. Comparative genomic analysis of KPC-encoding pKpQIL-like plasmids and their distribution in New Jersey and New York hospitals. Antimicrob Agents Chemother 58:2871–2877. doi:10.1128/AAC.00120-14 24614371 PMC3993205

[31] Partridge SR, Ginn AN, Wiklendt AM, Ellem J, Wong JSJ, Ingram P, Guy S, Garner S, Iredell JR. 2015. Emergence of bla_KPC_ carbapenemase genes in Australia. Int J Antimicrob Agents 45:130–136. doi:10.1016/j.ijantimicag.2014.10.006 25465526

[32] Arredondo-Alonso S, Willems RJ, van Schaik W, Schürch AC. 2017. On the (im)possibility of reconstructing plasmids from whole-genome short-read sequencing data. Microb Genom 3:e000128. doi:10.1099/mgen.0.000128 29177087 PMC5695206

[33] Hopkins KL, Ellaby N, Ellington MJ, Doumith M, Mustafa N, Meunier D, Woodford N. 2022. Diversity of carbapenemase-producing Enterobacterales in England as revealed by whole-genome sequencing of isolates referred to a national reference laboratory over a 30-month period. J Med Microbiol 71. doi:10.1099/jmm.0.001518 35604946

[34] Lee J-A, Du S-H, Lee T-F, Huang Y-S, Liao C-H, Huang Y-T, Hsueh P-R. 2023. Rapid emergence of ceftazidime-avibactam resistance among carbapenem-resistant Enterobacterales in a tertiary-care hospital in Taiwan. J Infect 86:66–117. doi:10.1016/j.jinf.2022.10.003 36257541

[35] Han M, Liu C, Xie H, Zheng J, Zhang Y, Li C, Shen H, Cao X. 2023. Genomic and clinical characteristics of carbapenem-resistant Enterobacter cloacae complex isolates collected in a Chinese tertiary hospital during 2013–2021. Front Microbiol 14:1127948. doi:10.3389/fmicb.2023.1127948 36896426 PMC9989974

[36] Zhou H, Wang S, Wu Y, Dong N, Ju X, Cai C, Li R, Li Y, Liu C, Lu J, Chan E-C, Chen S, Zhang R, Shen Z. 2022. Carriage of the mcr-9 and mcr-10 genes in clinical strains of the Enterobacter cloacae complex in China: a prevalence and molecular epidemiology study. Int J Antimicrob Agents 60:106645. doi:10.1016/j.ijantimicag.2022.106645 35907595

[37] Huang Y-S, Chen P-Y, Chou P-C, Wang J-T. 2023. In vitro activities and Inoculum effects of cefiderocol and aztreonam-avibactam against metallo-Β-lactamase-producing Enterobacteriaceae. Microbiol Spectr 11:e0056923. doi:10.1128/spectrum.00569-23 37154758 PMC10269523

[38] Hendrickx APA, Landman F, de Haan A, Borst D, Witteveen S, van Santen-Verheuvel MG, van der Heide HGJ, Schouls LM, Dutch CPE surveillance Study Group. 2020. Plasmid diversity among genetically related Klebsiella pneumoniae bla_KPC-2_ and bla_KPC-3_ isolates collected in the Dutch national surveillance. Sci Rep 10:16778. doi:10.1038/s41598-020-73440-2 33033293 PMC7546619

[39] Baraniak A, Izdebski R, Fiett J, Herda M, Derde LPG, Bonten MJM, Adler A, Carmeli Y, Goossens H, Hryniewicz W, Brun-Buisson C, Gniadkowski M, MOSAR WP2, WP3, and WP5 Study Groups. 2015. KPC-like carbapenemase-producing Enterobacteriaceae colonizing patients in Europe and Israel. Antimicrob Agents Chemother 60:1912–1917. doi:10.1128/AAC.02756-15 26711772 PMC4775924

[40] Jure MA, Castillo ME, Musa HE, López CG, Cáceres MA, Mochi SD, Bousquet AA, Genel NA, Arlet GA, Decré DC. 2019. Novel patterns in the molecular epidemiology of KPC-producing Klebsiella pneumoniae in Tucumán, Argentina. J Glob Antimicrob Resist 19:183–187. doi:10.1016/j.jgar.2019.02.015 30910742

[41] Findlay J, Hopkins KL, Loy R, Doumith M, Meunier D, Hill R, Pike R, Mustafa N, Livermore DM, Woodford N. 2017. OXA-48-like carbapenemases in the UK: an analysis of isolates and cases from 2007 to 2014. J Antimicrob Chemother 72:1340–1349. doi:10.1093/jac/dkx012 28199647

[42] León-Sampedro R, DelaFuente J, Díaz-Agero C, Crellen T, Musicha P, Rodríguez-Beltrán J, de la Vega C, Hernández-García M, López-Fresneña N, Ruiz-Garbajosa P, Cantón R, Cooper BS, San Millán Á, R-GNOSIS WP5 Study Group. 2021. Pervasive transmission of a carbapenem resistance plasmid in the gut microbiota of hospitalized patients. Nat Microbiol 6:606–616. doi:10.1038/s41564-021-00879-y 33782584 PMC7610705

[43] Macesic N, Hawkey J, Vezina B, Wisniewski JA, Cottingham H, Blakeway LV, Harshegyi T, Pragastis K, Badoordeen GZ, Dennison A, Spelman DW, Jenney AWJ, Peleg AY. 2023. Genomic dissection of endemic carbapenem resistance: metallo-beta-lactamase gene dissemination through clonal, plasmid and integron transfer pathways. bioRxiv. doi:10.1101/2023.03.25.534241 PMC10409761 37553339

[44] Andrade LN, Curiao T, Ferreira JC, Longo JM, Clímaco EC, Martinez R, Bellissimo-Rodrigues F, Basile-Filho A, Evaristo MA, Del Peloso PF, Ribeiro VB, Barth AL, Paula MC, Baquero F, Cantón R, Darini A da C, Coque TM. 2011. Dissemination of bla_KPC-2_ by the spread of Klebsiella pneumoniae clonal complex 258 clones (ST258, ST11, ST437) and plasmids (IncFII, IncN, IncL/M) among Enterobacteriaceae species in Brazil. Antimicrob Agents Chemother 55:3579–3583. doi:10.1128/AAC.01783-10 21576442 PMC3122403

[45] Bryant KA, Van Schooneveld TC, Thapa I, Bastola D, Williams LO, Safranek TJ, Hinrichs SH, Rupp ME, Fey PD. 2013. KPC-4 Is encoded within a truncated Tn4401 in an IncL/M plasmid, pNE1280, isolated from Enterobacter cloacae and Serratia marcescens. Antimicrob Agents Chemother 57:37–41. doi:10.1128/AAC.01062-12 23070154 PMC3535906

[46] Cuzon G, Naas T, Truong H, Villegas MV, Wisell KT, Carmeli Y, Gales AC, Venezia SN, Quinn JP, Nordmann P. 2010. Worldwide diversity of Klebsiella pneumoniae that produce β-lactamase bla_KPC-2_ gene. Emerg Infect Dis 16:1349–1356. doi:10.3201/eid1609.091389 20735917 PMC3294963

[47] Gomez SA, Pasteran FG, Faccone D, Tijet N, Rapoport M, Lucero C, Lastovetska O, Albornoz E, Galas M, KPC Group, Melano RG, Corso A, Petroni A. 2011. Clonal dissemination of Klebsiella pneumoniae ST258 harbouring KPC-2 in Argentina. Clin Microbiol Infect 17:1520–1524. doi:10.1111/j.1469-0691.2011.03600.x 21851480

[48] Eilertson B, Chen L, Chavda KD, Kreiswirth BN. 2017. Genomic characterization of two KPC-producing Klebsiella isolates collected in 1997 in New York City. Antimicrob Agents Chemother 61:e02458-16. doi:10.1128/AAC.02458-16 28167551 PMC5365676

[49] Gootz TD, Lescoe MK, Dib-Hajj F, Dougherty BA, He W, Della-Latta P, Huard RC. 2009. Genetic organization of transposase regions surrounding bla_KPC_ carbapenemase genes on plasmids from Klebsiella strains isolated in a New York City hospital. Antimicrob Agents Chemother 53:1998–2004. doi:10.1128/AAC.01355-08 19258268 PMC2681555

[50] Rada AM, De La Cadena E, Agudelo C, Capataz C, Orozco N, Pallares C, Dinh AQ, Panesso D, Ríos R, Diaz L, Correa A, Hanson BM, Villegas MV, Arias CA, Restrepo E. 2020. Dynamics of bla_KPC-2_ dissemination from non-CG258 Klebsiella pneumoniae to other Enterobacterales via IncN plasmids in an area of high endemicity. Antimicrob Agents Chemother 64:e01743-20. doi:10.1128/AAC.01743-20 32958711 PMC7674068

[51] Schweizer C, Bischoff P, Bender J, Kola A, Gastmeier P, Hummel M, Klefisch F-R, Schoenrath F, Frühauf A, Pfeifer Y. 2019. Plasmid-mediated transmission of KPC-2 carbapenemase in Enterobacteriaceae in critically Ill patients. Front Microbiol 10:276. doi:10.3389/fmicb.2019.00276 30837980 PMC6390000

[52] Hazen TH, Mettus R, McElheny CL, Bowler SL, Nagaraj S, Doi Y, Rasko DA. 2018. Diversity among bla_KPC_-containing plasmids in Escherichia coli and other bacterial species isolated from the same patients. Sci Rep 8:10291. doi:10.1038/s41598-018-28085-7 29980699 PMC6035167

[53] Tijet N, Muller MP, Matukas LM, Khan A, Patel SN, Melano RG. 2016. Lateral dissemination and inter-patient transmission of bla_KPC-3_: role of a conjugative plasmid in spreading carbapenem resistance. J Antimicrob Chemother 71:344–347. doi:10.1093/jac/dkv356 26518052

[54] Mataseje LF, Abdesselam K, Vachon J, Mitchel R, Bryce E, Roscoe D, Boyd DA, Embree J, Katz K, Kibsey P, Simor AE, Taylor G, Turgeon N, Langley J, Gravel D, Amaratunga K, Mulvey MR. 2016. Results from the Canadian nosocomial infection surveillance program on carbapenemase-producing Enterobacteriaceae, 2010 to 2014. Antimicrob Agents Chemother 60:6787–6794. doi:10.1128/AAC.01359-16 27600052 PMC5075087

[55] Stohr J, Verweij JJ, Buiting AGM, Rossen JWA, Kluytmans J. 2020. Within-patient plasmid dynamics in Klebsiella pneumoniae during an outbreak of a carbapenemase-producing Klebsiella pneumoniae. PLoS One 15:e0233313. doi:10.1371/journal.pone.0233313 32421705 PMC7233586

[56] Prussing C, Snavely EA, Singh N, Lapierre P, Lasek-Nesselquist E, Mitchell K, Haas W, Owsiak R, Nazarian E, Musser KA. 2020. Nanopore MinION sequencing reveals possible transfer of bla_KPC-2_ plasmid across bacterial species in two healthcare facilities. Front Microbiol 11:2007. doi:10.3389/fmicb.2020.02007 32973725 PMC7466660

[57] Martin J, Phan HTT, Findlay J, Stoesser N, Pankhurst L, Navickaite I, De Maio N, Eyre DW, Toogood G, Orsi NM, Kirby A, Young N, Turton JF, Hill RLR, Hopkins KL, Woodford N, Peto TEA, Walker AS, Crook DW, Wilcox MH. 2017. Covert dissemination of carbapenemase-producing Klebsiella pneumoniae (KPC) in a successfully controlled outbreak: long- and short-read whole-genome sequencing demonstrate multiple genetic modes of transmission. J Antimicrob Chemother 72:3025–3034. doi:10.1093/jac/dkx264 28961793 PMC5890743

[58] Neffe L, Abendroth L, Bautsch W, Häussler S, Tomasch J. 2022. High plasmidome diversity of extended-spectrum beta-lactam-resistant Escherichia coli isolates collected during one year in one community hospital. Genomics 114:110368. doi:10.1016/j.ygeno.2022.110368 35447310

[59] Barry KE, Wailan AM, Sheppard AE, Crook D, Vegesana K, Stoesser N, Parikh HI, Sebra R, Mathers AJ. 2019. Don’t overlook the little guy: an evaluation of the frequency of small plasmids co-conjugating with larger carbapenemase gene containing plasmids. Plasmid 103:1–8. doi:10.1016/j.plasmid.2019.03.005 30928702

[60] Viehweger A, Blumenscheit C, Lippmann N, Wyres KL, Brandt C, Hans JB, Hölzer M, Irber L, Gatermann S, Lübbert C, Pletz MW, Holt KE, König B. 2021. Context-aware genomic surveillance reveals hidden transmission of a carbapenemase-producing Klebsiella pneumoniae. Microb Genom 7:000741. doi:10.1099/mgen.0.000741 34913861 PMC8767333

[61] Bowers JR, Kitchel B, Driebe EM, MacCannell DR, Roe C, Lemmer D, de Man T, Rasheed JK, Engelthaler DM, Keim P, Limbago BM. 2015. Genomic analysis of the emergence and rapid global dissemination of the clonal group 258 Klebsiella pneumoniae pandemic. PLoS One 10:e0133727. doi:10.1371/journal.pone.0133727 26196384 PMC4510304

[62] Pallecchi L, Riccobono E, Mantella A, Fernandez C, Bartalesi F, Rodriguez H, Gotuzzo E, Bartoloni A, Rossolini GM. 2011. Small qnrB-harbouring ColE-like plasmids widespread in commensal enterobacteria from a remote Amazonas population not exposed to antibiotics. J Antimicrob Chemother 66:1176–1178. doi:10.1093/jac/dkr026 21393218

[63] Sugita K, Aoki K, Komori K, Nagasawa T, Ishii Y, Iwata S, Tateda K. 2021. Molecular analysis of bla_KPC-2_-harboring plasmids: Tn4401a interplasmid transposition and Tn4401a-carrying ColRNAI plasmid mobilization from Klebsiella pneumoniae to Citrobacter europaeus and Morganella morganii in a single patient. mSphere 6:e0085021. doi:10.1128/mSphere.00850-21 34730375 PMC8565517

[64] Moran RA, Hall RM. 2018. Evolution of regions containing antibiotic resistance genes in FII-2-FIB-1 ColV-Colla virulence plasmids. Microb Drug Resist 24:411–421. doi:10.1089/mdr.2017.0177 28922058

[65] Robertson J, Schonfeld J, Bessonov K, Bastedo P, Nash JHE. 2023. A global survey of Salmonella plasmids and their associations with antimicrobial resistance. Microb Genom 9:mgen001002. doi:10.1099/mgen.0.001002 37200081 PMC10272869

[66] Sheppard AE, Stoesser N, Wilson DJ, Sebra R, Kasarskis A, Anson LW, Giess A, Pankhurst LJ, Vaughan A, Grim CJ, Cox HL, Yeh AJ, Modernising Medical Microbiology (MMM) Informatics Group, Sifri CD, Walker AS, Peto TE, Crook DW, Mathers AJ. 2016. Nested Russian doll-like genetic mobility drives rapid dissemination of the carbapenem resistance gene bla_KPC_. Antimicrob Agents Chemother 60:3767–3778. doi:10.1128/AAC.00464-16 27067320 PMC4879409

[67] Orlek A, Stoesser N, Anjum MF, Doumith M, Ellington MJ, Peto T, Crook D, Woodford N, Walker AS, Phan H, Sheppard AE. 2017. Plasmid classification in an era of whole-genome sequencing: application in studies of antibiotic resistance epidemiology. Front Microbiol 8:182. doi:10.3389/fmicb.2017.00182 28232822 PMC5299020

[68] Lam MMC, Wick RR, Watts SC, Cerdeira LT, Wyres KL, Holt KE. 2021. A genomic surveillance framework and genotyping tool for Klebsiella pneumoniae and its related species complex. Nat Commun 12:4188. doi:10.1038/s41467-021-24448-3 34234121 PMC8263825

[69] Brehony C, McGrath E, Brennan W, Tuohy A, Whyte T, Brisse S, Maiden M, Jolley K, Morris D, Cormican M. 2019. An MLST approach to support tracking of plasmids carrying OXA-48-like carbapenemase. J Antimicrob Chemother 74:1856–1862. doi:10.1093/jac/dkz136 31225613 PMC6587408

[70] Pierce NT, Irber L, Reiter T, Brooks P, Brown CT. 2019. Large-scale sequence comparisons with sourmash. F1000Res 8:1006. doi:10.12688/f1000research.19675.1 31508216 PMC6720031

[71] Giménez M, Ferrés I, Iraola G. 2022. Improved detection and classification of plasmids from circularized and fragmented assemblies. bioRxiv. doi:10.1101/2022.08.04.502827

[72] Pradier L, Tissot T, Fiston-Lavier A-S, Bedhomme S. 2021. PlasForest: a homology-based random forest classifier for plasmid detection in genomic datasets. BMC Bioinformatics 22:349. doi:10.1186/s12859-021-04270-w 34174810 PMC8236179

[73] Wick RR, Judd LM, Gorrie CL, Holt KE. 2017. Unicycler: resolving bacterial genome assemblies from short and long sequencing reads. PLoS Comput Biol 13:e1005595. doi:10.1371/journal.pcbi.1005595 28594827 PMC5481147

[74] van Domselaar G. 2023. RefSeq Masher. National Microbiology laboratory. https://github.com/phac-nml/refseq_masher.

[75] Jain C, Rodriguez-R LM, Phillippy AM, Konstantinidis KT, Aluru S. 2018. High throughput ANI analysis of 90K prokaryotic genomes reveals clear species boundaries. Nat Commun 9:5114. doi:10.1038/s41467-018-07641-9 30504855 PMC6269478

[76] Bharat A, Petkau A, Avery BP, Chen JC, Folster JP, Carson CA, Kearney A, Nadon C, Mabon P, Thiessen J, Alexander DC, Allen V, El Bailey S, Bekal S, German GJ, Haldane D, Hoang L, Chui L, Minion J, Zahariadis G, Domselaar GV, Reid-Smith RJ, Mulvey MR. 2022. Correlation between phenotypic and in silico detection of antimicrobial resistance in Salmonella Enterica in Canada using Staramr. Microorganisms 10:292. doi:10.3390/microorganisms10020292 35208747 PMC8875511

[77] Zankari E, Hasman H, Cosentino S, Vestergaard M, Rasmussen S, Lund O, Aarestrup FM, Larsen MV. 2012. Identification of acquired antimicrobial resistance genes. J Antimicrob Chemother 67:2640–2644. doi:10.1093/jac/dks261 22782487 PMC3468078

[78] Seemann T. 2023. mlst. https://github.com/tseemann/mlst.

[79] Jolley KA, Bray JE, Maiden MCJ. 2018. Open-access bacterial population genomics: BIGSdb software, the PubMLST.org website and their applications. Wellcome Open Res 3:124. doi:10.12688/wellcomeopenres.14826.1 30345391 PMC6192448

[80] Cheruvanky A, Stoesser N, Sheppard AE, Crook DW, Hoffman PS, Weddle E, Carroll J, Sifri CD, Chai W, Barry K, Ramakrishnan G, Mathers AJ. 2017. Enhanced Klebsiella pneumoniae carbapenemase expression from a novel Tn4401 deletion. Antimicrob Agents Chemother 61:e00025-17. doi:10.1128/AAC.00025-17 28373185 PMC5444142

[81] Kitchel B, Rasheed JK, Patel JB, Srinivasan A, Navon-Venezia S, Carmeli Y, Brolund A, Giske CG. 2009. Molecular epidemiology of KPC-producing Klebsiella pneumoniae isolates in the United States: clonal expansion of multilocus sequence type 258. Antimicrob Agents Chemother 53:3365–3370. doi:10.1128/AAC.00126-09 19506063 PMC2715580

[82] Chen L, Mathema B, Chavda KD, DeLeo FR, Bonomo RA, Kreiswirth BN. 2014. Carbapenemase-producing Klebsiella pneumoniae: molecular and genetic decoding. Trends Microbiol 22:686–696. doi:10.1016/j.tim.2014.09.003 25304194 PMC4365952

[83] Araújo BF, Royer S, Campos PA, Ferreira ML, Gonçalves IR, Machado LG, Lincopan N, Fernandes MR, Cerdeira LT, Batistão D da F, Gontijo-Filho PP, Ribas RM. 2018. Insights into a novel Tn4401 deletion (Tn4401i) in a multidrug-resistant Klebsiella pneumoniae clinical strain belonging to the high-risk clonal group 258 producing KPC-2. Int J Antimicrob Agents 52:525–527. doi:10.1016/j.ijantimicag.2018.08.011 30165093

[84] Chmelnitsky I, Shklyar M, Leavitt A, Sadovsky E, Navon-Venezia S, Ben Dalak M, Edgar R, Carmeli Y. 2014. Mix and match of KPC-2 encoding plasmids in Enterobacteriaceae-comparative genomics. Diagn Microbiol Infect Dis 79:255–260. doi:10.1016/j.diagmicrobio.2014.03.008 24743043

[85] Tonkin-Hill G, MacAlasdair N, Ruis C, Weimann A, Horesh G, Lees JA, Gladstone RA, Lo S, Beaudoin C, Floto RA, Frost SDW, Corander J, Bentley SD. 2020. Producing polished prokaryotic pangenomes with the panaroo pipeline. Genome Biol 21:180. doi:10.1186/s13059-020-02090-4 32698896 PMC7376924

