# Supplementary figures and images for: Plasmid genomic epidemiology of blaKPC carbapenemase-producing Enterobacterales in Canada, 2010–2021
Source: Antimicrob Agents Chemother. 2023 Nov 16;67(12):e00860-23. doi: 10.1128/aac.00860-23 (PMC10720558; doi:10.1128/aac.00860-23)

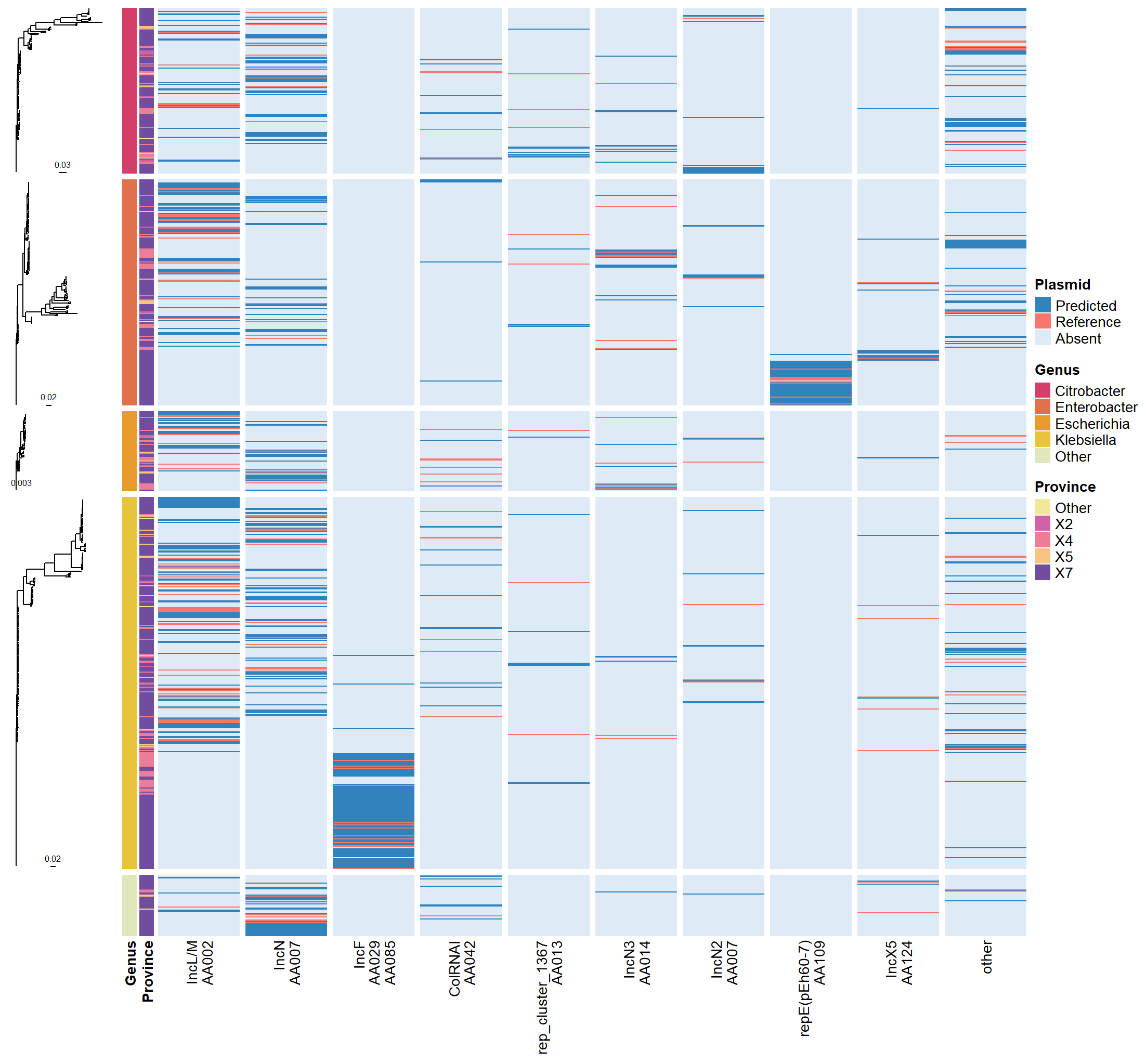

Supplement: Figure S1 — Prediction of blaKPC-encoding plasmids from incomplete assemblies using MOB-recon. [file aac.00860-23-s0001.tif]
