# Supplementary material for: Plasmid genomic epidemiology of blaKPC carbapenemase-producing Enterobacterales in Canada, 2010–2021
Source: Antimicrob Agents Chemother. 2023 Nov 16;67(12):e00860-23. doi: 10.1128/aac.00860-23 (PMC10720558; doi:10.1128/aac.00860-23)
Supplement: Additional method details [file aac.00860-23-s0002.docx]

SUPPLEMENTARY MATERIALS

*Canadian Nosocomial and Infection Surveillance Program isolates*

The Canadian Nosocomial Infection Surveillance Program (CNISP) is a sentinel surveillance system which collects epidemiological and linked microbiology data from 90 Canadian acute-care hospitals across 10 provinces and two territories. *Enterobacterales* organisms isolated from patients between 2010 and 2021 were eligible for inclusion by minimum inhibitory concentration (MIC) above clinical breakpoints [1] or if they tested positive using molecular (PCR) or phenotypic testing (mCIM, CARBA-NP) [2]. Serial isolates from the same patient were included if the organism or carbapenemase gene differed, and there were no restrictions on collection from particular body sites. Further information about the program can be found online (https://health-infobase.canada.ca/cnisp/).

Multiplex PCR to confirm carbapenemase gene bla_KPC_ was conducted as previously described [3]. We confirmed bla_KPC_ in 838 isolates, and early Shovill [4] assemblies with Illumina reads and StarAMR [5] further supported evidence of a *bla*_KPC_ gene in those isolates. However, upon assembling with the Unicycler [6] workflow used here, 9/838 isolates (about 1%) did not contain a bla_KPC_ gene in the final assembly. These are likely cases where the bla_KPC_ gene is found on a low copy number plasmid that it did not sequence well and could not be assembled consistently. To ensure consistency and replicability in our methods, these 9 isolates were excluded from our analyses but it is important to note that two programs (Shovill and Unicycler) which are both SPAdes wrappers could not consistently assemble our gene of interest from identical Illumina read sets in 1% of isolates. A total of 829 isolates encoding bla_KPC_ were collected from 2010 to 2021 from 34 hospitals and included in this study.

*Whole genome sequencing*

Genomic DNA was extracted using Epicentre MasterPure^TM^ Complete kits (Mandel Scientific, Guelph, ON, Canada). The same DNA extract was used for both short-read Illumina and long-read ONT sequencing where possible. Short-read libraries were created with TruSeq Nano DNA HT sample preparation kits (Illumina, San Diego, CA, USA). Paired-end, 301 bp indexed reads were generated on an Illumina MiSeq^TM^ platform (Illumina).

Long-read sequences were generated using the Rapid Barcoding Kit (SQK-RBK004) or the Rapid Barcoding Kit 96 (SQK-RBK110.96) on R9.4.1 flow cells with the MinION Mk1B (ONT, Oxford, Oxfordshire, UK). Read data was basecalled and demultiplexed with Guppy v6.3.7 using the Super High Accuracy model (ONT).

*Genome assemblies*

The assembly workflow was managed using Snakemake [7]. ONT reads were trimmed with Porechop v0.2.3_seqan2.1.1 [8] and filtered for Q-score > 8 and length > 1000 bases with Filtlong v0.2.1 [9]. Illumina reads had adaptors trimmed and were filtered for an average Q-score > 30 with trim-galore v0.6.7 [10]. FastQC v0.11.9 [11] and Nanoplot v1.28.2 [12] were used to assess quality control metrics for Illumina and ONT reads respectively. Illumina-only assemblies and hybrid assemblies with both ONT and Illumina reads were generated using Unicycler v0.5.0 [6]. Assemblies were polished with short reads using Polypolish v0.5.0 [13] and POLCA from MaSuRCA v4.0.9 [14].

*Organism identification and species complexes*

We used the following definitions for species complexes: The *K. pneumoniae* species complex includes *K. pneumoniae*, *K. quasipneumoniae*, and *K. variicola* [15]; the *Enterobacter cloacae* complex includes *E. cloacae*, *E. hormachei*, *E. asburiae*, *E. kobei*, and *E. ludwigii* [16]; and the *Citrobacter freundii* complex includes *C. freundii*, *C. portucalensis*, *C. werkmanii*, and *C. youngae* [17]. To assign species, Kleborate v2.2.0 [15] was used for species identification of *Klebsiella* with default parameters*.* Genomic clades and clusters in the *Enterobacter* spp. were defined by pairwise average nucleotide identity-based distance matrix using FastANI v1.3 [18], [19] and the clade was assigned when the mean average nucleotide identity value was > 95 %.

*Bioinformatic analyses and visualization*

Panaroo v1.3.2 [20] was used to estimate the pangenome and generate core gene alignments for each plasmid cluster as well as alignments for each of the top four genera (*Citrobacter, Enterobacter, Escherichia, Klebsiella*). The genus core gene alignments were converted to phylogenetic trees using FastTree v2.1.10 [21]. Bakta v1.6.1 [22] was used to annotate plasmids and PlasmidFinder v2.1 was used to investigate select replicons [23]. Plasmid taxonomic unit (PTU) designations were obtained from COPLA [24]. SNVPhyl [25] was used to investigate single nucleotide variants between *Enterobacter hormaechei* subsp. *hoffmannii* ST316 isolates using the following parameters: min_coverage = 10, min_mean_mapping = 30, SNV_abundance_ratio = 0.75, density threshold cutoff = 2, size of search window = 20.

Plots were created using R v4.3.0 [26] and the following packages: tidyverse packages [27], patchwork v1.1.2 [28], ComplexHeatmap v2.16.0 [29], [30], ggh4x v0.2.4 [31], RColorbrewer v1.1-3 [32], ggpubr v0.6.0 [33], ggtree v3.8.0 [34] and treeio v1.24.0 [35]. The plasmid map was created with Proksee [36], BLAST+ v2.12.0 with default parameters [37], and mobileOG db-1.6 [38] with default parameters (e-value: 1.0e-05, k_value: 1, query_score: 80, p_ident_value: 60).

*Plasmid clustering and containment analysis*

For plasmid clustering analysis, the PLSDB v2021_06_23v2 [39] database was downloaded and clustered alongside the 202 circular *bla*_KPC_-encoding plasmids in this study using MOB-cluster from the MOB-suite v3.1.4 package [40], [41]. MOB-cluster groups plasmids into primary and secondary clusters with pairwise Mash distances below specified thresholds (here using defaults 0.06 and 0.025) [40], [41]. In other words, all plasmids in each primary cluster have a Mash distance of < 0.06 from each other.

MOB-suite uses a Mash distance of 0.06 as a default as part of its complete linkage clustering approach which was optimized for Enterobacteriaceae; this threshold is permissive enough to allow plasmids of different sizes to be grouped together, which allows some change in sequence content that is characteristic of plasmids [40]. MOB-suite Mash distance-based clustering was compared to clustering with average nucleotide identity (FastANI) and the MOB-suite approach was found to perform better than identity metrics [41]. While some have criticized the 0.06 threshold as too stringent [42], a recent study [43] investigating *bla*_IMP-4_ carbapenemases clustered diverse plasmids from multiple isolates as we have done here, further supporting MOB-suite for plasmid investigation of diverse plasmid types.

For plasmid containment analysis of isolates with incomplete *bla*_KPC_ contigs, we used a combination of MOB-cluster and MOB-recon [40], [41] to predict plasmid content in isolates based on homology to existing plasmids in a custom database. All 1,856 circular plasmids completed in this study (including the 202 *bla*_KPC_-encoding plasmids) were used to create a custom Canadian plasmid database. Of those, 1,137 plasmids were completed from Illumina-only data (average size = 9.8 kb, median = 3.7 kb) and 719 were completed from ONT and Illumina hybrid assemblies (average size = 66.2 kb, median = 21.1 kb). All 829 isolates were screened for plasmids with MOB-recon using this custom database and the output was filtered to focus on the reconstructed plasmids containing *bla*_KPC_. MOB-recon reconstructs plasmids based on homology to plasmid markers (replicons, relaxases) and homology to plasmids in a database. Importantly, this approach cannot confirm *bla*_KPC_ presence in a particular plasmid nor can it capture rearrangements or insertions relative to the database plasmids; however, it does provide an indication of plasmid backbone prevalence and primary cluster membership across our isolate collection. Isolates with a complete *bla*_KPC_ plasmid were also submitted to MOB-recon as validation to confirm that the closest Mash neighbour in the reference plasmid database was itself.

SUPPLEMENTARY FIGURE LEGENDS AND TABLE CAPTIONS

**Supplementary Figure 1.** Prediction of *bla*_KPC_-encoding plasmids from incomplete assemblies using MOB-recon. “Predicted” indicates MOB-recon predicted that the incomplete *bla*_KPC_-encoding contig in that isolate is part of a plasmid that would group within that cluster. “Reference” indicates one of the 202 complete closed *bla*_KPC_-encoding plasmids we sequenced here. Whole genome maximum-likelihood trees were plotted for the four most common genera. The “Other” genera includes *Serratia* spp., *Raoultella* spp., *Pseudescherichia* spp., *Pantoea* spp., *Morganella* spp., *Kluyvera* spp. and *Hafnia* spp. Provinces were assigned an arbitrary number, and “Other” represents three provinces.

**Supplementary Table S1.** Isolate metadata and summary data

**Supplementary Table S2.** Complete *bla*_KPC_-encoding plasmid metadata and summary data

**Supplementary Table S3.** Updated Tn*4401* SNV table used in TETyper.

**Supplementary Table S4.** Updated Tn*4401* structural profiles used in TETyper.

REFERENCES

[1] Clinical and Laboratory Standards Institute, *Performance Standards for Antimicrobial Susceptibility Testing: Informational Supplement M100 ED33:2023*. Clinical and Laboratory Standards Institute, Wayne, PA, USA, 2023.

[2] Canadian Nosocomial Infection Surveillance Program, “Surveillance Protocol for Carbapenemase-Producing Organisms (CPO) in CNISP Hospitals.” Public Health Agency of Canada, 2023.

[3] L. F. Mataseje *et al.*, “Results from the Canadian Nosocomial Infection Surveillance Program on Carbapenemase-Producing Enterobacteriaceae, 2010 to 2014,” *Antimicrob. Agents Chemother.*, vol. 60, no. 11, pp. 6787–6794, Oct. 2016, doi: 10.1128/AAC.01359-16.

[4] T. Seemann, “Shovill.” May 09, 2023. Accessed: May 29, 2023. [Online]. Available: https://github.com/tseemann/shovill

[5] A. Bharat *et al.*, “Correlation between Phenotypic and In Silico Detection of Antimicrobial Resistance in Salmonella enterica in Canada Using Staramr,” *Microorganisms*, vol. 10, no. 2, Art. no. 2, Feb. 2022, doi: 10.3390/microorganisms10020292.

[6] R. R. Wick, L. M. Judd, C. L. Gorrie, and K. E. Holt, “Unicycler: Resolving bacterial genome assemblies from short and long sequencing reads,” *PLOS Comput. Biol.*, vol. 13, no. 6, p. e1005595, Jun. 2017, doi: 10.1371/journal.pcbi.1005595.

[7] F. Mölder *et al.*, “Sustainable data analysis with Snakemake.” Apr. 19, 2021. doi: 10.12688/f1000research.29032.2.

[8] R. Wick, “Porechop.” 2018. Accessed: Jan. 03, 2023. [Online]. Available: https://github.com/rrwick/Porechop

[9] R. Wick, “rrwick/Filtlong.” Dec. 30, 2022. Accessed: Jan. 03, 2023. [Online]. Available: https://github.com/rrwick/Filtlong

[10] F. Krueger, F. James, P. Ewels, E. Afyounian, and B. Schuster-Boeckler, “TrimGalore: v0.6.7.” Zenodo, Jul. 23, 2021. doi: 10.5281/zenodo.5127899.

[11] S. Andrews, “FastQC.” 2020. Accessed: Jan. 03, 2023. [Online]. Available: https://www.bioinformatics.babraham.ac.uk/projects/fastqc/

[12] W. De Coster, S. D’Hert, D. T. Schultz, M. Cruts, and C. Van Broeckhoven, “NanoPack: visualizing and processing long-read sequencing data,” *Bioinformatics*, vol. 34, no. 15, pp. 2666–2669, Aug. 2018, doi: 10.1093/bioinformatics/bty149.

[13] R. R. Wick and K. E. Holt, “Polypolish: Short-read polishing of long-read bacterial genome assemblies,” *PLOS Comput. Biol.*, vol. 18, no. 1, p. e1009802, Jan. 2022, doi: 10.1371/journal.pcbi.1009802.

[14] A. V. Zimin and S. L. Salzberg, “The genome polishing tool POLCA makes fast and accurate corrections in genome assemblies,” *PLoS Comput. Biol.*, vol. 16, no. 6, p. e1007981, Jun. 2020, doi: 10.1371/journal.pcbi.1007981.

[15] M. M. C. Lam, R. R. Wick, S. C. Watts, L. T. Cerdeira, K. L. Wyres, and K. E. Holt, “A genomic surveillance framework and genotyping tool for Klebsiella pneumoniae and its related species complex,” *Nat. Commun.*, vol. 12, no. 1, Art. no. 1, Jul. 2021, doi: 10.1038/s41467-021-24448-3.

[16] M. K. Annavajhala, A. Gomez-Simmonds, and A.-C. Uhlemann, “Multidrug-Resistant *Enterobacter cloacae* Complex Emerging as a Global, Diversifying Threat,” *Front. Microbiol.*, vol. 10, 2019, Accessed: Jun. 14, 2023. [Online]. Available: https://www.frontiersin.org/articles/10.3389/fmicb.2019.00044

[17] D. Brenner, P. Grimont, A. Steigerwalt, G. Fanning, A. Elisabeth, and C. Riddle, “Classification of Citrobacteria by DNA Hybridization: Designation of Citrobacter farmeri sp. nov., Citrobacter youngae sp. nov., Citrobacter braakii sp. nov., Citrobacter werkmanii sp. nov., Citrobacter sedlakii sp. nov., and Three Unnamed Citrobacter Genomospecies,” *Int. J. Syst. Bacteriol.*, vol. 43, pp. 645–58, Nov. 1993, doi: 10.1099/00207713-43-4-645.

[18] C. Jain, L. M. Rodriguez-R, A. M. Phillippy, K. T. Konstantinidis, and S. Aluru, “High throughput ANI analysis of 90K prokaryotic genomes reveals clear species boundaries,” *Nat. Commun.*, vol. 9, no. 1, Art. no. 1, Nov. 2018, doi: 10.1038/s41467-018-07641-9.

[19] G. G. Sutton, L. M. Brinkac, T. H. Clarke, and D. E. Fouts, “*Enterobacter hormaechei* subsp. *hoffmannii* subsp. nov., *Enterobacter hormaechei* subsp. *xiangfangensis* comb. nov., *Enterobacter roggenkampii* sp. nov., and *Enterobacter muelleri* is a later heterotypic synonym of *Enterobacter asburiae* based on computational analysis of sequenced *Enterobacter* genomes.,” *F1000Research*, vol. 7, p. 521, Jun. 2018, doi: 10.12688/f1000research.14566.2.

[20] G. Tonkin-Hill *et al.*, “Producing polished prokaryotic pangenomes with the Panaroo pipeline,” *Genome Biol.*, vol. 21, no. 1, p. 180, Jul. 2020, doi: 10.1186/s13059-020-02090-4.

[21] M. N. Price, P. S. Dehal, and A. P. Arkin, “FastTree 2 – Approximately Maximum-Likelihood Trees for Large Alignments,” *PLOS ONE*, vol. 5, no. 3, p. e9490, Mar. 2010, doi: 10.1371/journal.pone.0009490.

[22] O. Schwengers, L. Jelonek, M. A. Dieckmann, S. Beyvers, J. Blom, and A. Goesmann, “Bakta: rapid and standardized annotation of bacterial genomes via alignment-free sequence identification,” *Microb. Genomics*, vol. 7, no. 11, p. 000685, Nov. 2021, doi: 10.1099/mgen.0.000685.

[23] A. Carattoli *et al.*, “In Silico Detection and Typing of Plasmids using PlasmidFinder and Plasmid Multilocus Sequence Typing,” *Antimicrob. Agents Chemother.*, vol. 58, no. 7, pp. 3895–3903, Jul. 2014, doi: 10.1128/AAC.02412-14.

[24] S. Redondo-Salvo *et al.*, “COPLA, a taxonomic classifier of plasmids,” *BMC Bioinformatics*, vol. 22, no. 1, p. 390, Jul. 2021, doi: 10.1186/s12859-021-04299-x.

[25] A. Petkau *et al.*, “SNVPhyl: a single nucleotide variant phylogenomics pipeline for microbial genomic epidemiology,” *Microb. Genomics*, vol. 3, no. 6, p. e000116, Jun. 2017, doi: 10.1099/mgen.0.000116.

[26] R Core Team, “R: A language and environment for statistical computing.” R Foundation for Statistical Computing, Vienna, Austria, 2022. [Online]. Available: https://www.R-project.org/

[27] H. Wickham *et al.*, “ggplot2: Create Elegant Data Visualisations Using the Grammar of Graphics.” Apr. 03, 2023. Accessed: May 29, 2023. [Online]. Available: https://cran.r-project.org/web/packages/ggplot2/index.html

[28] T. L. Pedersen, “patchwork: The Composer of Plots.” Aug. 19, 2022. Accessed: Jun. 28, 2023. [Online]. Available: https://cran.r-project.org/web/packages/patchwork/index.html

[29] Z. Gu, R. Eils, and M. Schlesner, “Complex heatmaps reveal patterns and correlations in multidimensional genomic data,” *Bioinformatics*, vol. 32, no. 18, pp. 2847–2849, Sep. 2016, doi: 10.1093/bioinformatics/btw313.

[30] Z. Gu, “Complex heatmap visualization,” *iMeta*, vol. 1, no. 3, p. e43, 2022, doi: 10.1002/imt2.43.

[31] T. van den Brand, “ggh4x: Hacks for ‘ggplot2.’” Apr. 04, 2023. Accessed: May 29, 2023. [Online]. Available: https://cran.rstudio.com/web/packages/ggh4x/index.html

[32] E. Neuwirth, “RColorBrewer: ColorBrewer Palettes.” Apr. 03, 2022. Accessed: May 29, 2023. [Online]. Available: https://cran.r-project.org/web/packages/RColorBrewer/index.html

[33] A. Kassambara, “ggpubr: ‘ggplot2’ Based Publication Ready Plots.” Feb. 10, 2023. Accessed: May 29, 2023. [Online]. Available: https://cran.r-project.org/web/packages/ggpubr/index.html

[34] G. Yu, D. K. Smith, H. Zhu, Y. Guan, and T. T.-Y. Lam, “ggtree: an r package for visualization and annotation of phylogenetic trees with their covariates and other associated data,” *Methods Ecol. Evol.*, vol. 8, no. 1, pp. 28–36, 2017, doi: 10.1111/2041-210X.12628.

[35] L.-G. Wang *et al.*, “Treeio: An R Package for Phylogenetic Tree Input and Output with Richly Annotated and Associated Data,” *Mol. Biol. Evol.*, vol. 37, no. 2, pp. 599–603, Feb. 2020, doi: 10.1093/molbev/msz240.

[36] J. R. Grant *et al.*, “Proksee: in-depth characterization and visualization of bacterial genomes,” *Nucleic Acids Res.*, p. gkad326, May 2023, doi: 10.1093/nar/gkad326.

[37] S. F. Altschul, W. Gish, W. Miller, E. W. Myers, and D. J. Lipman, “Basic local alignment search tool,” *J. Mol. Biol.*, vol. 215, no. 3, pp. 403–410, Oct. 1990, doi: 10.1016/S0022-2836(05)80360-2.

[38] C. L. Brown *et al.*, “mobileOG-db: a Manually Curated Database of Protein Families Mediating the Life Cycle of Bacterial Mobile Genetic Elements,” *Appl. Environ. Microbiol.*, vol. 88, no. 18, pp. e00991-22, 2022, doi: 10.1128/aem.00991-22.

[39] G. P. Schmartz *et al.*, “PLSDB: advancing a comprehensive database of bacterial plasmids,” *Nucleic Acids Res.*, vol. 50, no. D1, pp. D273–D278, Jan. 2022, doi: 10.1093/nar/gkab1111.

[40] J. Robertson and J. H. E. Nash, “MOB-suite: software tools for clustering, reconstruction and typing of plasmids from draft assemblies,” *Microb. Genomics*, vol. 4, no. 8, p. e000206, Jul. 2018, doi: 10.1099/mgen.0.000206.

[41] J. Robertson, K. Bessonov, J. Schonfeld, and J. H. E. Nash, “Universal whole-sequence-based plasmid typing and its utility to prediction of host range and epidemiological surveillance,” *Microb. Genomics*, vol. 6, no. 10, p. mgen000435, Sep. 2020, doi: 10.1099/mgen.0.000435.

[42] S. Arredondo-Alonso *et al.*, “Consistent typing of plasmids with the mge-cluster pipeline.” bioRxiv, p. 2022.12.16.520696, Dec. 19, 2022. doi: 10.1101/2022.12.16.520696.

[43] N. Macesic *et al.*, “Genomic dissection of endemic carbapenem resistance: metallo-beta-lactamase gene dissemination through clonal, plasmid and integron transfer pathways.” bioRxiv, p. 2023.03.25.534241, Mar. 26, 2023. doi: 10.1101/2023.03.25.534241.
